# Biotechnological strategies and tools for *Plum pox virus* resistance: trans-, intra-, cis-genesis, and beyond

**DOI:** 10.3389/fpls.2015.00379

**Published:** 2015-06-08

**Authors:** Vincenza Ilardi, Mario Tavazza

**Affiliations:** ^1^Centro di Ricerca per la Patologia Vegetale, Consiglio per la Ricerca in Agricoltura e l’Analisi dell’Economia Agraria, Rome, Italy; ^2^UTAGRI Centro Ricerche Casaccia, Agenzia Nazionale per le Nuove Tecnologie, l’Energia e lo Sviluppo Economico Sostenibile, Rome, Italy

**Keywords:** *Potyvirus*, cisgenesis, intragenesis, eukaryotic translation initiation factors, DEAD-box RNA helicases, ZFN, TALEN, CRISPR/Cas9

## Abstract

*Plum pox virus* (PPV) is the etiological agent of sharka, the most devastating and economically important viral disease affecting *Prunus* species. It is widespread in most stone fruits producing countries even though eradication and quarantine programs are in place. The development of resistant cultivars and rootstocks remains the most ecologically and economically suitable approach to achieve long-term control of sharka disease. However, the few PPV resistance genetic resources found in *Prunus* germplasm along with some intrinsic biological features of stone fruit trees pose limits for efficient and fast breeding programs. This review focuses on an array of biotechnological strategies and tools, which have been used, or may be exploited to confer PPV resistance. A considerable number of scientific studies clearly indicate that robust and predictable resistance can be achieved by transforming plant species with constructs encoding intron-spliced hairpin RNAs homologous to conserved regions of the PPV genome. In addition, we discuss how recent advances in our understanding of PPV biology can be profitably exploited to develop viral interference strategies. In particular, genetic manipulation of host genes by which PPV accomplishes its infection cycle already permits the creation of intragenic resistant plants. Finally, we review the emerging genome editing technologies based on ZFN, TALEN and CRISPR/Cas9 engineered nucleases and how the knockout of host susceptibility genes will open up next generation of PPV resistant plants.

## Introduction

The *Prunus* genus (family *Rosaceae*) includes many fruit crop species, commonly named stone fruits, widely grown due to the organoleptic characteristics and nutritional value of their drupes (Potter, 2012). These species are of great economic importance reaching in 2013 the worldwide production of 43.7 million tons (MT) composed by 21.6 MT of peaches (*P. persica*) and nectarines (*P. persica* var. *nucipersica*), 11.5 MT of plums (*P. domestica* and *P. salicina*), and sloes (*P. spinosa*), 4.1 MT of apricots (*P. armeniaca*), 2.9 MT of almonds with shell (*P. dulcis*, syn. *P. amygdalus*), 2.3 MT of sweet cherries (*P. avium*), and 1.3 MT of sour cherries (*P. cerasus*) as reported cherries by FAO (FAOSTAT^1^).

The most devastating and economically important disease affecting stone fruits is plum pox, also named sharka (García et al., 2014; Barba et al., 2015). It causes severe fruit symptoms such as malformations, ringspots and in some cases premature drop. The infection affects the ripening process, especially fruit coloration, softening and weight, and significantly modifies the composition of nutritive and phenolic compounds (Usenik et al., 2015). Sharka was first detected in Eastern Europe in the early 1900s. From there it has spread to most European countries, around the Mediterranean basin, and now, despite quarantine regulations (e.g., European and Mediterranean Plant Protection Organization^2^, North American Plant Protection Organization’s^3^), the disease has been reported in most stone fruit producing countries worldwide except Australia, New Zealand, California (USA), and South Africa (CABI datasheet, 2014). Even today, plants affected by sharka disease are occasionally intercepted in internationally traded *Prunus* planting material. The costs related to sharka involve both direct losses associated with decreased fruit quality and yield, eradication of infected plants and compensatory measures, and indirect costs related to preventative measures and control strategies (Cambra et al., 2006).

**FIGURE 1:**
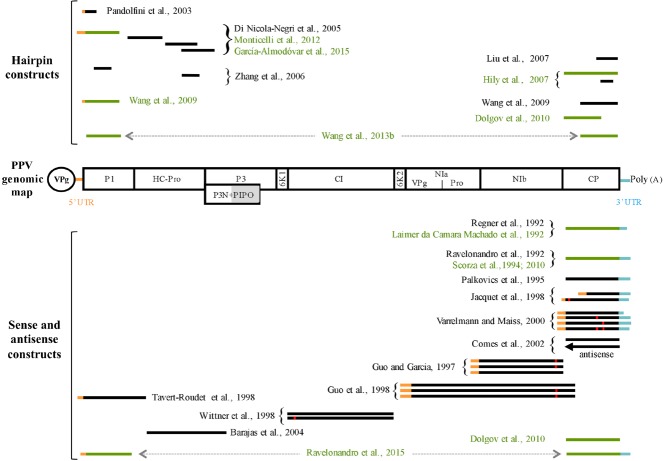
**Genomic map of *Plum pox virus* (PPV) and schematic representation of PPV sequences utilized for obtaining transgenic PPV-resistant plants.** The long viral open reading frame is represented by a box divided into different proteins, named accordingly. PIPO protein, which is expressed after a frameshift, is indicated by a grey box below the P3 region. The viral protein linked to the genome (VPg) is represented as an ellipse. PPV sequences utilized for obtaining transgenic plants are schematized as horizontal lines above (hairpin constructs) or below (sense or antisense constructs) the PPV map. The black lines refer to the constructs tested only in model plants. The green lines refer to construct used to transform also stone fruit plants with the exception of Dolgov et al. (2010) who transformed only plum. The orange and light blue lines portions correspond to the PPV 5′ and 3′ UTR regions, respectively. Red squares on lines indicate the presence of mutated nucleotide sequences. PPV sequences connected by a double arrow were arranged in the same molecular construct.

The etiological agent of sharka is *Plum pox virus* (PPV), genus *Potyvirus* (García et al., 2014; Revers and García, 2015). The PPV genome consists of a single-stranded, positive-sense RNA, of about 9,770 nucleotides (nt) in length, encapsidated by a single coat protein (CP) in a rod-shaped particle. The genomic RNA carries a covalently linked virus-encoded protein (VPg) at its 5′ end and poly (A) tail at its 3′ end. It contains a long open reading frame that is translated into a large polyprotein precursor from the second AUG codon (García et al., 2014). The polyprotein undergoes proteolytic processing catalyzed by three viral-encoded proteinases to produce at least 10 mature protein products. In addition, another protein, P3N-PIPO, is potentially produced by translational frameshift (Figure 1). Like all phytoviruses, PPV is an obligate intracellular parasite possessing a limited genome capacity. Therefore, to accomplish its infection cycle, PPV relies on the multifunctional properties of its proteins and on the contribution of a diverse array of host factors (García et al., 2014; Revers and García, 2015).

*Plum pox virus* is transmitted by aphids in a non-persistent manner. A single probe of a viruliferous aphid is sufficient to inoculate in a receptor GF305 peach seedling an average of about 26,000 PPV RNA molecules, with a 20% chance of resulting in a systemic infection (Moreno et al., 2009). In addition, PPV is transmitted by grafting but it does not seem to be transmitted through pollen and seeds (Pasquini and Barba, 2006). Thus, PPV spreads locally by aphids if infected plants are present in orchards and over long distance by using infected vegetative propagation material.

*Plum pox virus* isolates which possess specific symptomatology, host range, epidemiology, pathogenicity, genome sequences and aphid transmissibility have been grouped into at least eight monophyletic strains: D, M, Rec, T, EA, W, C and CR (García et al., 2014). Among the PPV strains M, D and Rec are the most important and widespread.

Viral diseases cannot be directly controlled by chemical application on infected plants. PPV control strategies are based on the use of certified PPV-free plant material, periodic surveys of orchards, eradication of diseased trees and on insecticide treatment to control aphid populations. However, the reduction of aphids by insecticide treatment has only a limited success to contain viruses, such as PPV, which are transmitted in a non-persistent manner (Perring et al., 1999).

Taking into account the above data, the development of PPV-resistant stone fruit remains the best approach to control PPV. However, despite screening efforts, few sources of PPV resistance have been identified into the *Prunus* germplasm. In particular, PPV-resistance was found in *P. davidiana* clone P1908 a peach closely related species (Rubio et al., 2010), in a few North American apricot genotypes (Marandel et al., 2009), in almond (Rubio et al., 2003), and in some plum clones characterized by PPV-induced hypersensitive response (HR; Hartmann and Neumuller, 2009). Unfortunately, in addition to the reduced and not well molecularly characterized PPV-resistance traits other constraints such as incompatibility barriers between species, the slow and challenging selection process due to long generation times and the high degree of heterozygosis have posed serious limits for efficient and fast breeding programs. All these findings have prompted the scientific community to explore the use of genetic engineering technologies as additional tools to develop *Prunus* varieties and rootstocks resistant to PPV.

Transgenesis refers to the genetic engineering approach that modifies a recipient plant by introducing DNA sequences isolated from any eukaryotic (excluding crossable, sexually compatible, organisms), prokaryotic, virus and viroid, as well as *de novo* synthesized sequences (EFSA panel on Genetically Modified Organisms GMO, 2012). When the genetic elements are from crossable organisms, then it is called intragenesis or cisgenesis (see the section on intragenesis and cisgenesis for sharka resistance, below). In addition, new targeted-mutagenesis technologies based on engineered nucleases have been recently developed, which promise to revolutionize future plant genetic engineering.

This review focuses on the biotechnological researches conducted in the last 10 years and the recent advances and future perspectives that can be envisaged for producing PPV resistant plants.

## Transformation and Regeneration of *Prunus* Species: From Conundrum to Reality

The production of trans-, intra-, or cis-genic plants all require, as the first step, genome modification of a plant cell followed by its regeneration in a whole organism. Taking into account the elevated heterozygosis of *Prunus* species, the best-suited explants to be targeted are adult plant materials and not seed-derived ones (e.g., embryos, immature cotyledons, or mature seed hypocotyls) as the latter do not produce clonal sources of regenerants.

Till a few years ago, the production of transgenic *Prunus* was confined to a few species and mostly obtained from juvenile plant material because fruit trees are particularly recalcitrant to regeneration from mature explants (Petri and Burgos, 2005). The efficiency and robustness of the transformation/regeneration processes were far from that established in several annual plants. For this reason, PPV resistance strategies were previously tested on easier to transform *Nicotiana* species. In particular, the *N. benthamiana* has been extensively used for proof-of-concept demonstration of new biotechnological strategies devoted to controlling PPV infection (Ravelonandro et al., 1992, 2015; Regner et al., 1992; Palkovics et al., 1995; Guo and Garcia, 1997; Guo et al., 1998; Jacquet et al., 1998; Tavert-Roudet et al., 1998; Wittner et al., 1998; Pandolfini et al., 2003; Barajas et al., 2004; Di Nicola-Negri et al., 2005; Zhang et al., 2006; Hily et al., 2007; Liu et al., 2007; Wang et al., 2013b).

In the last years, refined and detailed protocols for the production of transgenic *Prunus* starting from adult plant materials, such as shoot tips of peach (Sabbadini et al., 2015) and leaf explants of apricot (Petri et al., 2015), almonds (Ramesh et al., 2006), and cherry (Song, 2015), have been published. The transformation efficiency being between 0.3 and 5.6% based on the species/genotype analyzed. In addition, a high transformation efficiency protocol (up to 42%) for the plum ‘Bluebyrd’ starting from mature seed hypocotyl slices has been set up (Petri et al., 2008). This result poses the ‘Bluebyrd’ as the *Prunus* “model” plant for biotechnological studies.

Although the transformation/regeneration protocols of *Prunus* species still need to be improved, there is no doubt that they have opened the way for the genetic improvement of *Prunus* species through biotechnology. Notably, once a single transgenic plant with the desired characteristic is obtained, such as the plum clone C5 (see the next paragraph), vegetative propagation will result in an unlimited production of the transgenic plant.

## Transgenesis: a Robust Approach to Confer PPV Resistance

Most of the transgenic strategies developed to control virus infection are based on the use of sequences derived from the viral genome. In particular, from the early 1990s till the discovery of RNA silencing, the prevailing idea was that ectopic expression of wild-type or opportunely mutagenized viral proteins, as well as the expression of viral antisense RNAs, could interfere with the viral life cycle. From a conceptual point of view, all these initial strategies can be envisaged as the exploitation of the pathogen-derived resistance (PDR) concept developed by Sanford and Johnston (1985). Coherently, the molecular approaches developed in the first 10 years of the PPV resistance genetic engineering era (1992–2002) substantially follow the scheme described above, in that several PPV genome sequences were exploited to confer resistance (Figure 1 and references therein; Ilardi and Di Nicola-Negri, 2011). Although these transgenic plants were designed to express different PPV derived proteins, they can be substantially considered all together from a mechanistic point of view. In fact, common aspects of this first generation of transgenic plants are: (i) the resistance did not positively correlate with the amount of transgenic RNA/protein accumulating in the transgenic plants, (ii) in several resistant plants, transgenic proteins were undetectable, (iii) only a minor fraction of the transgenic plants was resistant, and (iv) a fraction of the transgenic plants recovers with time after viral infection. Retrospectively, it is clear that the resistant phenotypes were not protein-mediated but were the result of the unpredictable activation of the RNA silencing pathway stimulated by the integration, in the host genome, of multiple/rearranged transgene copies which in turn led to transcription of aberrant or double-stranded RNAs (dsRNA).

RNA silencing refers to a family of sequence-specific gene silencing phenomena by which the expression of nucleic acid sequences is downregulated or entirely suppressed (for reviews see, Baulcombe, 2004; Brodersen and Voinnet, 2006; Vaucheret, 2008; Axtell, 2013; Bologna and Voinnet, 2014; Matzke and Mosher, 2014). The triggers are partially or wholly dsRNAs, which are recognized and diced by host Dicer-like enzymes into short molecules (small interfering RNAs, siRNAs) of 21–24 nucleotides in length. The siRNAs are then protected from degradation by 2′-O-methylation and loaded onto a multi-subunit RNA-Induced Silencing Complex (RISC), which, in the case of post-transcriptional gene silencing (PTGS), guides sequence-specific degradation of homologous RNAs. In addition, RNA-dependent RNA polymerases, in particular, RDR6 together with SGS3, amplify the RNA silencing response by turning aberrant RNAs into dsRNA, the triggering molecule (Mourrain et al., 2000; Qu et al., 2008). RNA silencing is an ancient mechanism, which in plants, among other functions, has a significant role in defending the host from viruses (Ding and Voinnet, 2007). As counterattack, viruses encode proteins, called viral suppressors of RNA silencing (VSRs), able to interfere with different steps of the RNA silencing pathway (for review see, Csorba et al., 2015).

Among the PPV resistant transgenic plants produced during the early efforts the most studied and successful is the plum clone C5, now renamed ‘HoneySweet’ and approved for cultivation in the USA (reviewed, in Scorza et al., 2013a). ‘Stanley’ plum was transformed with the sense PPV-D *CP* gene and among several transgenic lines obtained, only C5 showed PPV resistance under greenhouse conditions (Scorza et al., 1994). From a molecular point of view, C5 possesses multiple and rearranged *CP* gene copies, expresses low level of *CP* mRNA and does not accumulate detectable amount of CP (Scorza et al., 1994, 2001). *CP* transgene is methylated, and *CP* specific siRNAs are produced (Scorza et al., 2001). All these findings indeed indicate that C5 PPV resistance is the result of RNA silencing activation against the PPV *CP* sequence. In order to confirm this hypothesis an inserted copy of the *CP*, rearranged as an inverted repeat, was cloned from C5 and transferred into ‘Bluebyrd’ plum seedlings. The aphid mediated infection test performed on these new transgenic plants demonstrated that this rearranged *CP* sequence is able, in some clones, to provide resistance to a PPV-D isolate (Scorza et al., 2010).

The C5 clone was extensively evaluated in field studies for over 10 years to validate its ability to resist PPV infection under natural environmental conditions. Plantings were made in Poland, Spain, Romania and the Czech Republic at sites characterized by endemic sharka presence (Malinowski et al., 2006; Polak et al., 2008). C5 plants were resistant to PPV when exposed to natural viruliferous aphids while when graft-inoculated, accumulated low level of PPV, mostly near the graft junction. C5 plants were graft-inoculated with different combinations of viruses affecting stone fruits to test the efficacy and stability of PPV resistance. In particular, *Prunus necrotic ring spot virus* (PNRSV), *Apple chlorotic leaf spot virus* (ACLSV), *Prunus dwarf virus* (PDV), and PPV-D (Zagrai et al., 2008) and PPV-Rec (Polak et al., 2008) were used in these studies. All together, these analyses confirmed that: (i) C5 is highly resistant to PPV infection, and low level of PPV can only accumulate in some grafted plants, and (ii) heterologous virus infection sustained by PNRSV, ACLSV, or PDV do not suppress PPV resistance. In addition, an 11-year study performed in West Virginia, USA, assessed that gene flow was quite low depending on the distance and environmental conditions (Scorza et al., 2013b). Based on the positive results obtained with the C5 clone, Ravelonandro et al. (2015) recently transformed plum plants with a gene construct containing the 5′ region of the PPV genome fused to the 3′ one (Figure 1). Similarly to what observed with the C5 construct, the two resistant plum clones do not produce CP but accumulate specific siRNAs, the hallmark of gene silencing activation.

Although important results were occasionally obtained with some sense construct, like C5, the principal limitation of this strategy is the impossibility to introduce, in a predictable way, the resistance trait to the same or different species using the same molecular construct. Therefore, once understood that PTGS was the mechanism underlying most, if not all, transgenic PPV resistance and that virus-derived gene constructs encoding intron-spliced hairpin RNAs (ihpRNAs) can efficiently induce PTGS (Smith et al., 2000), several constructs expressing PPV-derived ihpRNAs were developed (Figure 1). All the new ihpRNA constructs were designed starting from viral isolates belonging to PPV-D or PPV-M strain and selecting highly conserved genomic regions, at the nucleotide level, among different PPV strains (Figure 1). This choice was due to the fact that: (i) viral isolates of PPV-D and PPV-M strains are the most economically important ones, and (ii) typically, RNA silencing confers resistance to viral isolates sharing more than 90% of sequence identity with the introduced transgenes (Maki-Valkama et al., 2000; Bau et al., 2003; Xu et al., 2009) even though only 21 nt of perfect homology between the hairpin construct and the RNA target can be sufficient to confer resistance (Lu et al., 2004).

The first PPV ihpRNA construct was developed by Pandolfini et al. (2003; Figure 1). They transformed the model plant *N. benthamiana* with a short PPV-D derived sequence covering the AUG translation initiation codon under the transcriptional control of the *rolC* promoter. As expected by the phloem specificity of the promoter, systemic but not local PPV resistance was obtained. In a successive work, Di Nicola-Negri et al. (2005) showed that CaMV 35 S-driven expression of ihpRNAs covering the 5′UTR/*P1*, *P1/HC-Pro*, *HC-Pro*, or *HC-Pro/P3* sequences (Figure 1) confers efficient and predictable resistance to the homologous PPV-M isolate. Notably, *N. benthamiana* plants transformed with the h-UTR/P1 construct were also highly-resistant/immune to seven PPV isolates belonging to D, M, and Rec strains (Di Nicola-Negri et al., 2010). In addition, plant lines expressing high levels of h-UTR/P1-derived siRNAs were also resistant to the PPV-EA and PPV-C isolates, which share an overall nucleotide identity with the transgene of only 77.8 and 71.2%, respectively (Di Nicola-Negri et al., 2010; Di Nicola et al., 2014). Interestingly, h-UTR/P1-plants grown at low (15°C) or high (30°C) temperatures, which are known to potentially affect PTGS and virus resistance (Szittya et al., 2003; Wu et al., 2008; Zhong et al., 2013), were fully resistant to multiple PPV challenges, different PPV inoculum concentrations, and the distantly related PPV-C isolate (Di Nicola et al., 2014). Moreover, pre-infection of h-UTR/P1-plants with a virus belonging to *Cucumovirus*, *Potyvirus*, or *Tombusvirus*, all known to encode VSRs able to interfere with the PTGS pathway, does not affect PPV resistance (Di Nicola et al., 2014). On the basis of the results obtained with the model plant, two EU laboratories used the h-UTR/P1 construct to transform plum. Transgenic plum clones were resistant to distinct PPV-D isolates (Monticelli et al., 2012; García-Almodóvar et al., 2015). The ability of P1 and HC-Pro ihpRNA constructs (Figure 1) to confer PPV resistance was further confirmed by Zhang et al. (2006).

In order to build a single construct able to interfere with the most important stone fruit-infecting viruses, Liu et al. (2007) produced an ihpRNA construct fusing sequences from six stone fruit infecting viruses including, among them, a portion of a PPV-D *CP* gene (Figure 1). Of the twenty-eight transgenic *N. benthamiana* lines obtained, two T3 homozygous ones were selected for multi-virus resistance tests. T3 plants challenged with a PPV-D isolate did not show any visible symptoms. Unfortunately, the size of the construct seems to affect the ability to transform cherry plants (Song et al., 2013). In a work of the same period, Hily et al. (2007) transformed *N. benthamiana* plants with ihpRNA constructs containing the full-length or the second half of the PPV *CP* gene under the transcriptional control of either CaMV 35S or peach *Cab* promoters (Figure 1). The full-length CP construct driven by the CaMV 35S promoter outperformed all the other constructs, and for this reason was chosen for plum transformation. Transgenic plums were tested over multiple cycles of vegetative growth with PPV isolates belonging to D, M, Rec, C, and EA strains. Most of the ihpRNA-CP plants were resistant to all viral isolates. A few ihpRNA-CP plants failed to prevent PPV replication only in the areas close to the grafting point (Ravelonandro et al., 2014).

In another work, Wang et al. (2009) developed two ihpRNA constructs from a Canadian PPV-D isolate using either the 5′ portion of the *P1* gene or the 3′portion of the *CP* one. Similarly to the previous works, the construct that gave the best resistance results (ihpRNA P1) in the model plant was chosen to transform plum. Transgenic plum lines were challenged with a Canadian PPV-D isolate via chip-budding. PPV was undetectable in five out of the 10 T0 transgenic lines analyzed. In the wake of these results, the same research group produced a triple-intron-spanned double-hairpin RNA construct, which simultaneously targets PPV *P1* and *CP* sequences. This construct outperforms the previous ones in conferring PPV resistance in both *N. benthamiana* and plum plants (Wang et al., 2013b).

Collectively, these data confirm the robustness of the ihpRNA-mediated PPV resistance. In fact, even though RNA silencing operates in a sequence-specific fashion, reports from different laboratories clearly show that it is possible to select PPV viral sequences capable to confer a robust, broad spectrum of resistance to most, if not all, PPV strains (Di Nicola-Negri et al., 2010; Di Nicola et al., 2014; Ravelonandro et al., 2014).

RNA-mediated virus resistance can also be brought about by the expression of opportunely engineered microRNAs (miRNAs). miRNAs are a class of small RNAs that play a substantial role in regulating gene expression (for reviews see, Mallory and Vaucheret, 2006; Voinnet, 2009; Axtell, 2013). Plant miRNAs, which are 19–24 nt in length are generated from processing of longer precursors, successively the pri-miRNA and the pre-miRNA. The mature miRNA is recruited to the RISC complex to downregulate, in a sequence-specific manner, their target mRNAs. Importantly, it is possible to modify a pri-miRNA/pre-miRNA sequence in such a way that the mature miRNA results complementary to a desired RNA target. Thus, using this approach, it is possible to build artificial miRNA (amiRNA) capable to target any sequence.

The first evidence that natural miRNA can target a plant virus was demonstrated by Simón-Mateo and García (2006), which showed that PPV chimeras bearing wild-type, but not mutated, miR171, miR167, and miR159 target sequences have an impaired infectivity on *N. benthamiana* and *Arabidopsis thaliana* plants. In the same year, Niu et al. (2006) transformed *A. thaliana* plants with engineered pre-miRNAs capable to generate mature amiRNAs complementary to the *P69* gene of *Turnip yellow mosaic virus* (TYMV) or the *HC-Pro* gene of *Turnip mosaic virus* (TuMV) or both. *A. thaliana* plants engineered to express these amiRNAs were specifically resistant to TYMV, TuMV or both, depending on the construct(s) used. Similar strategies based on the expression of one, two or more amiRNAs conferred resistance to: *Cucumber mosaic virus* (CMV) in *N. tabacum* (Qu et al., 2007), *A. thaliana* and tomato (Zhang et al., 2011); *Potato virus Y* in *N. tabacum* (Jiang et al., 2011); *Watermelon silver mottle virus* in *N. benthamiana* (Kung et al., 2012); and *Wheat streak mosaic virus* in wheat (Fahim et al., 2012).

The above examples show the feasibility of using the amiRNA technology to confer PPV resistance. In principle, one advantage of using amiRNAs instead of ihpRNAs is that it should be possible to design an efficient amiRNA without having off-target effects (Jackson et al., 2003). Off-target effects refer to unintended host gene regulation effects that may occur as a result of sequence homology between siRNAs or amiRNAs and mRNAs of the recipient organism (Senthil-Kumar and Mysore, 2011). Conversely, ihpRNA technology is superior to the amiRNA one in respect of the stability of the resistance trait. In fact, it was shown that viral RNA genomes could quickly evolve thus escaping amiRNA-mediated targeting (Lin et al., 2009; Lafforgue et al., 2011; Martínez et al., 2012). To overcome this inconvenient amiRNAs targeting distinct viral genome regions or amiRNA targeting highly conserved viral sequences should be expressed in the plant (Lafforgue et al., 2013). The above aspect clearly assumes a minor relevance with respect to standard ihpRNA constructs, which generate a pool of diverse siRNA molecules complementary to an extended (>400 nt) virus genomic region.

Mixed viral infection can be envisaged as an additional cause potentially affecting amiRNA-mediated resistance. In fact, VSRs have been shown to interfere not only with PTGS but also with the miRNA pathway (Kasschau et al., 2003). In a recent work, Martínez et al. (2013) analyzed the fate of the amiRNA-mediated resistance to TuMV challenging the *A. thaliana* plants with viruses belonging to five different taxonomic groups in addition to TuMV. TuMV resistance was overcome when plants were pre-infected with the *Tobacco rattle virus* (TRV), *Cauliflower mosaic virus*, or CMV but not with TYMV, PPV, or the *Lettuce mosaic potyvirus*. These results suggest that preinfection with some viruses other than potyviruses can jeopardize amiRNA-mediated resistance. In the context of sharka resistance, it will be appropriate to test the impact of mixed viral infections with PNRSV, ACLSV, and PDV (most common *Prunus*-infecting viruses) on amiRNAs functionality.

Although antibodies are a part of the vertebrate adaptive immune system and are not present in plants, a pioneering work showed that ectopic expression in *N. benthamiana* of an engineered antibody fragment (scFv) directed to recognize the CP of the *Artichoke mottled crinkle virus* confers resistance to the homologous virus (Tavladoraki et al., 1993). Successively, resistance to multiple viruses was achieved expressing scFvs recognizing a conserved protein domain of the plant viruses RNA-dependent RNA polymerase (Boonrod et al., 2004). The scFv strategy was recently exploited to confer PPV resistance in *N. benthamiana* (Gil et al., 2011). In particular, a scFV specific to the NIb RNA replicase was expressed in different plant cellular compartments. Between 8 and 84% of the transgenic T1 plants challenged with a moderate PPV inoculum dose was virus free. Thus, scFV-mediated PPV resistance appears not as robust as that induced by ihpRNAs or amiRNAs, and additional work will be required to understand whether this strategy can be profitably used to fully control PPV infection.

## Intragenesis and Cisgenesis for PPV Resistance

About 10 years ago, two new plant biotechnological breeding techniques, intragenesis and cisgenesis, were proposed (Rommens, 2004; Schouten et al., 2006). They were both devoted to overcoming one of the major public concerns related to the release of transgenic plants into the environment, i.e., introducing genetic material evolutionary distantly related to the recipient plant. The common unifying concept behind intragenesis and cisgenesis is that the genetic material present in the final modified plant can only derive from plant species within the sexual compatibility pool. In particular, whereas in intragenesis mixing of regulatory sequences (e.g., promoters and terminators) derived from different genes as well as the introduction of mutations (e.g., nucleotide substitutions, sequence deletions, duplication and inversions) is allowed, in cisgenic plants a native complete gene sequence with all its regulatory regions, comprised of introns, is used (Holme et al., 2013). A direct corollary of the above approaches is that selectable marker genes such as those conferring resistance to antibiotics (e.g., *hpt* and *nptII*) or herbicides (e.g., *bar*) should not be used or should be removed after plant transformation (Yau and Stewart, 2013). In this context, the high efficiency transformation of the plum’ ‘Bluebyrd’ allows the regeneration of marker-free plants using the non-selected transformation approach (Petri et al., 2011), whereas a site-specific recombinase-mediated approach was used to obtain marker-free transgenic apricots (López-Noguera et al., 2009; Petri et al., 2012).

At first glance, it could be counterintuitive that intragenesis and cisgenesis actually represent a powerful tool to control sharka disease because few PPV resistant characters have been identified in *Prunus* species and the genes behind these resistance traits are still not unambiguously characterized (Rubio et al., 2010, 2013, 2014; Soriano et al., 2012; Zuriaga et al., 2013; Decroocq et al., 2014).

However, in addition to the genetic variability present in nature, rearrangements and mutations of plant gene sequences open the way for developing new virus interference strategies. Notably, the deep dependence of virus infectivity on host factors defines the susceptibility host genes as an attractive pool where to fish for resistance, and molecular, genetic and genomic tools are now available for their identification. In particular, beside plant-induced mutagenesis (Lellis et al., 2002) interaction studies among viral and host proteins represent a robust approach for identifying new potential host susceptibility factors (Huang et al., 2010; Elena and Rodrigo, 2012). The rationale is based upon the idea that in a non-negligible number of cases the interaction underlines the requirement of the host factor to accomplish or aid the execution of a viral infection step such as translation, replication or movement. Consequently, host gene knockout or knockdown or mutations affecting its capacity to bind the viral protein could result in the impairment of virus infectivity.

In nature, the virus resistant trait of a mutated susceptibility gene usually comes apparent in homozygosis thus being, from a genetic point of view, a recessive character. Most of the natural recessive plant genes involved in virus resistance encode for the eukaryotic translation initiation factors 4E (eIF4E), 4G (eIF4G) or their isoforms, eIF(iso)4E and eIF(iso)4G (Wang and Krishnaswamy, 2012). Interestingly, recessive resistances against *Potyvirus* appear to occur more frequently than in other virus genera (Truniger and Aranda, 2009).

eIF4E exerts an essential role during the initiation of the eukaryotic mRNA translation by interacting with the 5′-terminal cap structure, this interaction being a limiting step for an efficient translation (Jackson et al., 2010). As reported above, the 5′ terminus of *Potyvirus* genomic RNA is covalently linked to the VPg but not to the cap structure. However, pioneering experiments showed that TuMV VPg interacts with *A. thaliana* eIF(iso)4E (Wittmann et al., 1997). In addition, mutation of a single amino acid in the TuMV VPg domain that binds eIF(iso)4E abolishes virus ability to infect *Brassica perviridis* plants (Léonard et al., 2000) and ethyl methanesulfonate induced mutations of *A. thaliana eIF(iso)4E* gene confer recessive resistance to TuMV (Lellis et al., 2002). In the following years, several *Potyvirus* natural recessive resistance genes linked to *eIF4E* or *eIF(iso)4E* were identified (Ruffel et al., 2002; Wang and Krishnaswamy, 2012). Depending on the virus and plant species studied mutations in *eIF4E*, *eIF(iso)4E*, or in both genes are required to confer recessive resistance. Importantly, knockout and knockdown experiments suggest that, at least under normal growth conditions, altered eIF4E functions can be compensated by eIF(iso)4E and *vice-versa* giving rise to viable plants phenotypically indistinguishable from wild-type ones (Duprat et al., 2002; Wang et al., 2013a).

In the context of sharka resistance, *P. davidiana* clone P1908, a species closely related to peach, is characterized by a complex polygenic PPV resistance character involving at least six quantitative trait *loci* three of which co-localize in genomic regions where gene copies of *eIF4E* and *eIF(iso)4G* exist (Marandel et al., 2009; Rubio et al., 2010). However, whether *eIF4E* or *eIF(iso)4G* are involved in this resistance trait remain to be elucidated. Conversely, previous studies showed that disruption of *A. thaliana eIF(iso)4E*, by transposon tagging, confers resistance to PPV isolates of D, M, C, and EA strains (Decroocq et al., 2006). This data strongly supports the absolute requirement of this susceptibility factor for PPV infectivity. Recently, Wang et al. (2013a) cloned *P. domestica* genes encoding eIF(iso)4E and eIF4E showing that the first, but not the latter protein interacts with PPV VPg. Intron-hairpin constructs of plum *eIF4E* and *eIF(iso)4E* were independently introduced in plum plants. Transgenic plants harboring *eIF(iso)4E* hairpin but not *eIF4E* one were resistant to a PPV-D isolate (Wang et al., 2013a). In addition, based on the previous PPV resistance results (Decroocq et al., 2006) it is expected that these plum plants are also resistant to PPV isolates of M, C, and EA strains. Thus, eIF(iso)4E was required for PPV infectivity both in *A. thaliana* and in *P. domestica*. This behavior differs from that observed in the case of *Tobacco etch virus*, which uses eIF(iso)4E to infect *A. thaliana* but uses eIF4E to infect *Capsicum annum* (Estevan et al., 2014).

Notably, silencing of the *eIF(iso)4E*, as well as of any recessive host susceptibility genes, confers a dominant PPV resistant trait permitting its ready transmission by crossing. In addition, when multiple copies of a susceptibility gene are present per haploid genome the RNA silencing approach results remarkably superior to classical breeding for fixing recessive resistant traits. In fact, a hairpin construct in heterozygous conditions can silence all the multiple copies of the susceptibility gene resulting in a full resistance. Thus, manipulation of susceptibility host genes, for conferring PPV resistance, is already a reality. The next step in developing a PPV resistant intragenic line is to adopt the same or similar silencing strategies (e.g., amiRNA) but using regulatory sequences (e.g., promoters and terminators) derived from a *Prunus* sexually compatible plant and avoiding the use of selectable markers. An additional strategy to confer PPV resistance is to overexpress an opportunely modified version of the plum eIF(iso)4E protein. A similar approach has been already utilized with success in potato (Cavatorta et al., 2011).

The above data also suggest that host proteins that interfere with eIF(iso)4E could be potential candidates for PPV resistance. In this context, Castelló et al. (2010) showed that the *A. thaliana* DNA-binding protein phosphatase 1 (AtDBP1) interacts with and stabilizes eIF(iso)4E protein. When *atdp1* plants were challenged with a GFP-tagged version of PPV, a 40-fold lower accumulation of viral RNA was observed. Further experiments of the same research group identified an additional host susceptibility protein named GRF6, which interacts with both AtDBP1 and the mitogen-activated protein kinase 11 (MPK11; Carrasco et al., 2014). How GRF6 promotes PPV susceptibility is unknown. However, knockout experiments show a 12-fold reduction of PPV accumulation in *grf6* plants. Identification and functional analyses of AtDBP1 and GRF6 *Prunus* orthologs are necessary to evaluate whether a silencing approach of these genes can be profitably used to control sharka disease.

Huang et al. (2010) using the yeast two-hybrid system identified the *P. persica* DEAD-box RNA helicase-like (PpDDXL) and the *A. thaliana* DEAD-box RNA helicase AtRH8 as additional interactors of VPg. AtRH8 and PpDDXL VPg-binding regions are highly conserved, and the interactions with VPg were confirmed to occur in plant. Importantly, PPV was not able to infect a knockout mutant of *AtRH8* indicating the close requirement of AtRH8 for a successful virus infection. Notably, *atrh8* plants did not show developmental differences with respect to wild-type plants thus suggesting that knockdown of *PpDDXL* expression through RNA silencing should result in PPV resistant viable peach plants. In addition, transient overexpression of AtRH8 and PpDDXL deletion mutants retaining the VPg-binding region reduces PPV accumulation by 3- to 5-fold (Huang et al., 2010). Thus, in addition to the silencing approach, the overexpression of a PpDDXL dominant negative mutant could be used to confer PPV resistance. It remains to be demonstrated whether stable expression of this dysfunctional product completely prevents virus infection. The theoretical advantage of using such a dominant negative mutant is that the same construct can be used for different peach cross-compatible species without the requirement to clone and to functionally characterize other *Prunus* VPg-interacting DEAD-box RNA helicase genes.

Virus-host protein interaction studies performed on a susceptible host can identify, in addition to the host factors required for a successful infection, genes with an antiviral role. In this case, the interaction can be envisaged as a way for the virus to counterattack, escape or attenuate plant defense responses. In this context, the PPV cylindrical inclusion (CI) protein was shown to interact with the PSI-K subunit of *N. benthamiana* photosystem I. The steady-state level of the psaK mRNA, which encodes PSI-K, decreases in PPV inoculated leaves. In addition, coexpression of CI negatively impacts transiently expressed PSI-K, and, importantly, transgenic *N. benthamiana* silenced for *psaK* were more susceptible to PPV (Jiménez et al., 2006). These data suggest that PSI-K has an antiviral role. It would be of interest to know whether *Prunus* PSI-K orthologs interact with PPV CI and have a similar role in plant defense. In another study, the *A. thaliana mpk11* loss-of-function mutant was shown to enhance the susceptibility to PPV (Carrasco et al., 2014). MPK11 appears to exert its PPV antiviral role by promoting GRF6 degradation. Thus, both PSI-K and MPK11 deserve future attention as potential candidates for intragenic and cisgenic approaches.

In addition to the host factors identified by studying PPV–host interactions, the molecular characterization of the few PPV resistance genetic traits of pomological value present in some cultivars of *P. armeniaca* (e.g., ‘Goldrich’ and ‘Harcot’; Dondini et al., 2011; Zuriaga et al., 2013; Decroocq et al., 2014), *P. domestica* (e.g., ‘Jojo’; Hartmann and Neumuller, 2009) and *P. dulcis* (e.g., ‘Garrigues’; Rubio et al., 2013) could offer, in a future, an opportunity for developing new resistance strategies. Although the genetic resistance to PPV in *P. armeniaca* is still controversial with one, two or three genes being responsible for it, a major resistance *locus* (*PPVres*) present in the linkage group 1 has been sequenced, and the number of the candidate genes was restricted to 23 (Zuriaga et al., 2013). In particular, a cluster of six meprin and TRAF-C homology (MATHd) domain containing proteins has been suggested as the best candidates for PPV resistance (Zuriaga et al., 2013). However, recent work shows that the presence of *PPVres* is not sufficient to unambiguously confer PPV resistance, thus supporting the notion that at least another *locus* should be involved (Decroocq et al., 2014). Thus, additional work is required to identify the gene harbored by this extra *locus* and to establish whether MATHd proteins can be profitably used alone or together with the former for introducing PPV resistance in *Prunus* species.

Even though the molecular characterization of the PPV HR of the plum ‘Jojo,’ which is oligogenically controlled (Hartmann and Neumuller, 2009) is still in its infancy, this resistant trait deserves a particular interest. In fact, HR is often the outcome of the direct or indirect recognition of a plant resistance (R) gene with a pathogen avirulence factor, and the largest class of the R genes encode nucleotide-binding leucine-rich repeat (NB-LRR) proteins (Dangl and Jones, 2001). A recent work shows that 354 NB-LRR genes are expressed in ‘Jojo’ plants undergoing PPV-induced HR and that 10 of them were differentially expressed respect the uninfected control plants (Rodamilans et al., 2014). Whether NB-LRR proteins are behind the ‘Jojo’ resistance is not known. However, in the case that NB-LRRs are involved in this phenotype then they can be profitably exploited to confer a robust PPV resistance in *Prunus* species. In fact, pioneering works showed that: (i) virus resistance mediated by NB-LRR proteins can be transferred between different species (Bendahmane et al., 1999), (ii) the spectrum of viral isolates recognized by NB-LRR can be broadened through artificial evolution (Farnham and Baulcombe, 2006), and (iii) the trade-off of costs and benefits resulting from the ectopic expression of modified NB-LRR genes can be opportunely modulated (Harris et al., 2013).

A recent work shows that grafting the PPV-D resistant ‘Garrigues’ almond onto the PPV susceptible peach GF305 before virus inoculation prevents, in almost the totality of GF305 challenged plants, virus infection (Rubio et al., 2013). The ability of the ‘Garrigues’ to transmit, through a graft junction, the resistance phenotype to GF305 prompts the authors to point out to a diffusible factor(s) as responsible for the observed resistance. Among the potential host factors, the authors speculate on the *RTM* (Restricted *Tobacco etch virus* Movement) proteins. The *A. thaliana RTM* genes are atypical dominant R genes that restrict the long distance movement (LDM) of some potyvirus and do not encode NB-LRR proteins. In particular, some PPV-EA and PPV-M but not PPV-D isolates show a restricted LDM (Decroocq et al., 2006, 2009). Infection analysis using recombinant PPV genomes indicate in the first 146 N-terminal amino acid of CP the virus determinant involved in overcoming RTM-mediated resistance (Decroocq et al., 2009). *RTM1* encode a jacalin-type lectin, RTM2 a small heat shock protein while *RTM3* a MATHd protein. Mutations in each of the three dominant *RTM* genes abolish the resistance suggesting that they act together probably in a multiprotein complex. Coherently, RTM1 and 2 are expressed in phloem-associated tissues (Chisholm et al., 2001) and RTM 1 and 3 were shown to interact (Cosson et al., 2010). Recently, two other RTM *loci* were identified (Cosson et al., 2012). Thus, at least five dominant *RTM* genes concur in the LDM resistance phenotype. Although the mechanism of ‘Garrigues’ resistance is still far from to be characterized and up today there is no evidence for the involvement of *RTM*-like genes, its characterization will be useful for the development of an additional PPV resistance strategy.

From the above examples it appears clear that while some intragenic approaches based on the silencing of host susceptibility genes such as *eIF(iso)4E* and *PpDDXL* are already a close reality, others need to be confirmed in *Prunus* species (e.g., orthologs of *AtDP1* and *GRF6*), and others will be available in the future as soon as the molecular and functional characterization of the few resistant characters found in *Prunus* will be disclosed.

## Plant Genome Editing: A Bright Future for PPV Resistance

In the last years, new targeted-mutagenesis technologies based on engineered nucleases have been developed, which promise to revolutionize biological and applied research fields spanning from agriculture to personalized medicine (Gaj et al., 2013; Fichtner et al., 2014; Cox et al., 2015). In particular, clustered regulatory interspaced short palindromic repeats (CRISPR)/Cas-based endonucleases (Jiang et al., 2013; Nekrasov et al., 2013; Shan et al., 2013a), transcription activator-like effector nucleases (TALENs; Li et al., 2012; Shan et al., 2013b; Zhang et al., 2013), and zinc-finger nucleases (ZFNs; Shukla et al., 2009; Townsend et al., 2009; Osakabe et al., 2010) have paved the way for efficient plant genome editing. Genome editing technologies permit to introduce in complex genomes gene-specific modifications such as deletions, insertions as well as gene replacement.

CRISPR/Cas9, TALEN, and ZFN nucleases share a common fundamental principle that is the ability to introduce a DNA double-stranded break (DSB) in almost any sequence of interest regardless of its function (Gaj et al., 2013). Importantly, the introduced DSB stimulates the host cellular DNA repair machinery that is the instrument through which the mutations are introduced. In somatic cells, DNA repair follows two conserved different routes, the error-prone non-homologous end-joining (NHEJ) pathway and the homologous recombination (HR). NHEJ operates throughout the entire cell cycle whereas HR is mainly active during S and G_2_ phases. Thus, the error-prone NHEJ is the principal DNA repair mechanism operating in the somatic cells (Knoll et al., 2014).

In eukaryotes at least two NHEJ mechanisms exist. In the canonical NHEJ (cNHEJ) pathway, a certain proportion of the DSBs is religated restoring the original sequence. However, frequent deletions or insertion of few nucleotides occur at the junction site. In addition, a mutagenic alternative NHEJ (aNHEJ) pathway operates together with the cNHEJ in repairing DSBs (Knoll et al., 2014). The aNHEJ pathway frequently causes deletions of several nucleotides at the junction site. In the second DNA repair route (HR pathway), a DNA template is used to repair the DSB. In natural conditions, this template is the sister chromatid or the homologous chromosome. However, if an exogenous DNA template sharing sequence homology with the regions flanking the DSB is provided, the final repaired DNA sequence can contain the exogenous DNA. Collectively, when a single DSB is introduced into the genome the DNA repair machinery can lead to gene knockout due to frameshift mutations and large deletions, which are mostly introduced by the NHEJ pathway, or if an exogenous DNA template is provided a precise gene replacement or gene insertion can be obtained. Thus, differently from the classical mutagenesis (e.g., EMS, gamma ray), which induces random mutations in the genome, the engineered nucleases promised to precisely mutagenize only the desired target gene(s).

**FIGURE 2:**
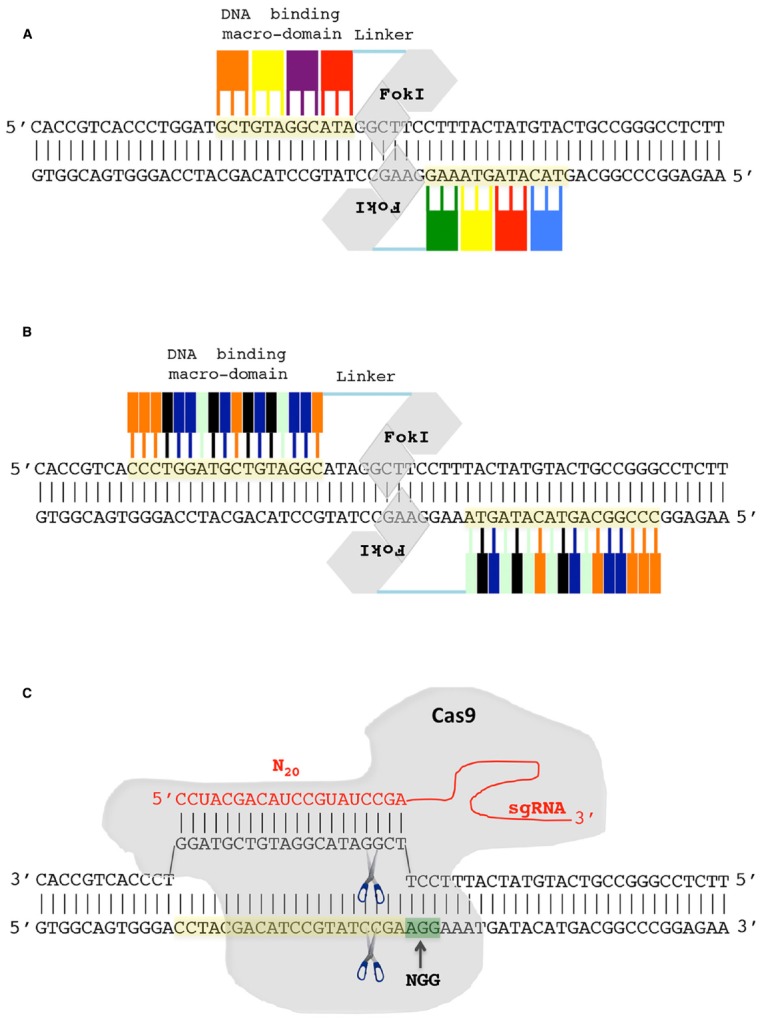
**Genome editing using engineered nucleases. (A)** Zinc-finger nuclease (ZFN). A pair of ZFNs is required to introduce a double-strand break (DSB). The ZFN DNA binding macro-domain is composed of subdomain each recognizing three nucleotides. The FoKI endonuclease domain of ZFN introduces, after dimerization, the DSB. **(B)** Transcription activator-like effector nuclease (TALEN). The TALEN DNA binding macro-domain is composed of subdomains each recognizing a single nucleotide. Similarly to ZFN a pair of TALENs is required to introduce a FoKI-mediated DSB. **(C)** In CRISPR/Cas9 system the first 20 nucleotides of engineered single-guide RNA (sgRNA) guides the nuclease Cas9 to recognize and cut the DNA target. The NGG nucleotides (green box), DNA target (yellow box).

From a schematic point of view, the engineered nucleases can be described as composed of two modules, a variable sequence-specific recognition module, which is opportunely engineered to recognize the selected DNA target, and a conserved nuclease one that executes the DSB (Figure 2). The two modules are fused in a single protein in both ZFNs and TALENs. In particular, ZFN and TALEN target recognition is mediated by ZF and TALE DNA binding macro domain, respectively, while, the nuclease module is, for both proteins, the non-specific nuclease domain of the restriction endonuclease *FoK*I. Importantly, dimerization of the *FoK*I nuclease domain is required for DNA cleavage (Bitinaite et al., 1998; Wah et al., 1998). Therefore, to introduce a DSB, a pair of ZFNs or TALENs proteins, which recognize sequences on the opposite DNA strands and positioned in proximity to each other in a such a way to make possible the dimerization of *FoK*I nuclease domain, is required (Figures 2A,B). DSB occurs in the spacer sequences between the two opposite ZFN and TALEN binding sites. The spacer length depends on the length of the linker protein connecting the binding macro domain with the FokI domain (Händel et al., 2009; Mussolino et al., 2011). In the most recently engineered ZFNs a short linker protein is used that require a spacer of five-six bp (Händel et al., 2009) while for TALENs the spacer is between 12 and 22 bp (Miller et al., 2011; Mussolino et al., 2011). The ZF DNA binding macro domain is normally composed of 3–4 ZFs subunits each of which consists of approximately 30 amino acid and able to specifically recognize one of nearly all the 64 possible three nucleotides combinations (Figure 2A). Thus, a typical ZFN DNA target is 9–12 nucleotide in length. Similarly to ZF, the TALE DNA binding macro domain is composed of subunits. However, in this case, each subunit recognize a single base pairs and not a group of three nucleotides (Figure 2B). More than 20 subunits can be arranged thus giving high flexibility and specificity in DNA target recognition.

In the last years, ZFNs and TALENs have been profitably used to create targeted-specific mutations in model and crop plants (Shukla et al., 2009; Townsend et al., 2009; Osakabe et al., 2010; Li et al., 2012; Shan et al., 2013b) even though in some cases they can suffer from some limitations. In particular, ZF subdomains can interfere each other impairing the ability to target a predicted sequence and high level of ZFN expression can result in cell toxicity (Cornu et al., 2008; Ramirez et al., 2008; Pruett-Miller et al., 2009). On the other side, TALEN *in vitro* DNA binding activity not always corresponds to that observed *in vivo* (Meckler et al., 2013). The recently exploited CRISPR/Cas9 tool overcomes the criticalities of ZFNs and appears more easily to handle than TALEN system if a large-scale mutagenesis project is undertaken (see Fichtner et al., 2014, for a comparison of pros and cons of ZFNs, TALENs and CRISPR/Cas9 systems). In particular, in CRISPR/Cas9 system the two modules, DNA binding and nuclease, are physically separated (Figure 2C). DNA sequence recognition is achieved by base complementarity with the first 20 nucleotides of an engineered RNA molecule called the single guided RNA (sgRNA) whereas the Cas9 endonuclease introduces the DSB. Importantly, the sgRNA associates with and allows Cas9 to specifically recognize and cleave the DNA. The only additional requirement for the CRISPR/Cas9 system is the presence of the NGG sequence adjacent to the 3′ end of the 20 base pairs target (N_20_-NGG). Thus, any sequence of the form N_20_-NGG can be targeted by the CRISPR/Cas9 system simply by engineering the sgRNA in such a way that its first 20 nucleotides are complementary to the N_20_-NGG target sequence (Figure 2C). The physical separation between the recognition module and the nuclease one, and the easy way to reprogram the DNA binding specificity make CRISPR/Cas9 a flexible tool for genome editing (Li et al., 2013; Shan et al., 2013a; Feng et al., 2014).

In the more recurrent scheme, the so-called in planta genome engineering, transgenic plants harboring the ZFN, TALEN, or CRISPR/Cas9 nuclease are recovered after transformation of plant explants, protoplasts or embryonic cells (Li et al., 2012; Nekrasov et al., 2013). In this case, gene targeting occurs during plant life-span. Genetic segregation and molecular analysis of the offspring allow the recovery of non-transgenic plants, which are specifically mutated in the selected host gene (Feng et al., 2014). However, genetic segregation can be less affordable for economically important *Prunus* species characterized by a high degree of heterozygosis and a long juvenile stage. For example, under field condition the juvenile phase of almond, cherry, peach and plum is between three and seven years depending on the species take into consideration (Hansche, 1986; Besford et al., 1996; García-Gusano et al., 2010; Srinivasan et al., 2012). A possible strategy to overcome the long reproductive cycle would be to transform *Prunus* species with a transgene construct containing in addition to the engineered nuclease(s) a flowering promoting gene such as the *Flowering Locus T* 1 (*FT1*) gene. In a recent work, Srinivasan et al. (2012) showed that transgenic plums, highly expressing the poplar *FT1* gene, flower and produce fruits within 1–10 months. Thus, the proposed strategy that couple engineered nucleases with a flowering promoting gene appears feasible to achieve targeted mutagenesis in *Prunus* species. Alternatively, the nucleases need to be expressed transiently in such a way that the final plants would contain only the mutated host gene. A promising approach consists in using virus expression vectors to transiently express the engineered nuclease. Marton et al. (2010) engineered TRV to express in planta a ZFN. Using this approach they were able to recover petunia and tobacco plants opportunely mutagenized but ZFN-free. Due to the limited cargo capacity and/or the nature of its genomic material (RNA virus) this vector can be used for ZFN but not TALEN or CRISPR/Cas9 delivering. A further technological breakthrough in this field was the demonstration that a deconstructed geminivirus vector expressing a ZFN together with a DNA repair molecule enhances DNA targeting by more than one order of magnitude (Baltes et al., 2014). These results advocate the use of plant virus vectors for efficient plant genome editing.

Which host genes could be edited to confer sharka resistance? As reported above the error-prone NHEJ mostly induces insertions and deletions in absence of a DNA repair molecule. Thus, if a nuclease is engineered to recognize and cut a DNA sequence closely positioned to the translation start codon, the outcome will be the knockout of the gene. Taking into consideration this aspect, the host susceptibility genes represent, up to now, the best candidates to confer sharka resistance. In particular, as previously discussed, *eIF(iso)4E* and *PpDDXL* can be profitably manipulated for conferring PPV resistance. In fact, knockout of *eIF(iso)4E* (Decroocq et al., 2006) or *AtRH8* (Huang et al., 2010) in *A. thaliana* and knockdown of *eIF(iso)4E* in plum all confer resistance to PPV (Wang et al., 2013a). In addition to gene knockout of the host susceptibility genes as soon as we will be able to characterize PPV resistance genes (e.g., almond HR-promoting genes) these could be introduced by gene replacement in the susceptible species/cultivar.

Although up to now no genome-edited *Prunus* species have been reported, there is no doubt that this technology will assume, in a future, a great importance for stone fruits genetic improvement in general and sharka resistance in particular. In fact, although intragenesis technology already offers the opportunity to confer resistance to PPV by knocking down the host susceptibility genes, public concerns over the cultivation of such plants could prevent its widespread application. In particular, the presence of extra gene sequences randomly integrated into the host genome in both intra- and cisgenic plants have been envisaged as a potential hazard. Conversely, in the case of NHEJ-mediated gene knockout no extra copies of DNAs are present, and the final plant is comparable with those arising from natural mutations. However, although the advantages in term of safety, robustness and speed of the precision genome editing technologies over the classical mutagenesis are apparent far beyond the circle of plant researchers their wide application will require the development of dedicated and harmonized legislations. In particular, it is desirable that the resulting plant trait and not the methodology used would be at the basis of the new legislations. In this direction are the recent scientific conclusions of a panel of experts consulted by the Food Standards of Australia and New Zealand: “*where targeted mutagenic techniques are used to introduce small, site-specific mutations involving only one or a few nucleotides, and any transgenes have been segregated away from the final food producing lines, derived food products would be similar to food produced using traditional mutagenic techniques and should not be regarded as GM food*^4^.”

## Concluding Remarks

Research over the last 10 years has provided compelling evidences on the important contribute that biotechnology can offer to obtain *Prunus* species resistant to sharka disease. Notably, the production of transgenic plums resistant to PPV is already e reality (Hily et al., 2007; Wang et al., 2009, 2013a,b; Scorza et al., 2010; Monticelli et al., 2012; García-Almodóvar et al., 2015) and the first engineered clone ‘HoneySweet’ has been cleared for cultivation in the USA (Scorza et al., 2013a). In addition, our better understanding of the interaction between PPV and its model host (*A. thaliana*) has opened the way for the initial identification of the plant molecular network utilized by the virus to accomplish its infection cycle (Bosque et al., 2014; García et al., 2014). Although this network could differ in some respects from that present in a *Prunus* species, it is expected that highly interconnected host proteins, similarly required by others potyviruses, should be conserved among different plant species. Thus, the identification of such hub proteins represents a future research objective for developing new interference strategies. The eIF(iso)4E is a clear example of that (Decroocq et al., 2006; Wang et al., 2013a).

Highly accurate genome sequence of the double haploid peach ‘Lovell’ has been recently reported (Verde et al., 2013^5^) In addition, the recent development of the third generation sequencing technologies with their capability to sequence long individual molecules of DNA coupled with the high throughput and base call accuracy of second generation ones will give an important contribute to unveil the complexity of polyploidy *Prunus* genomes (Faino and Thomma, 2014). The availability of *Prunus* genome sequences will boost the stone fruit research in general and genome editing in particular. In this context, the development of dedicated engineered *Prunus*-infecting viruses to transiently express engineered nucleases represents an important future research area. In fact, the realization of such new tools promise to speed up and simplify the creation of genome-edited *Prunus* species, possibly overcoming the bottleneck of the transformation-regeneration process. Moreover, the availability of *Prunus* genome sequences will help the isolation and functional characterization of stone fruits regulative gene sequences (e.g., promoters, terminators, introns and UTRs) required to develop intragenic constructs.

In addition, a biotechnological approach that deserves future attention is the use of intragenic *Prunus* rootstocks silenced for a susceptibility host gene. In fact, it is expected that, under certain conditions, the RNA silencing signal, moving from the intragenic rootstock to a non-transgenic scion, would be able to silence the susceptibility gene in scion tissues thus conferring PPV resistance. The knowledge and the technical aspects to test this hypothesis are already available. In fact, protocols to obtain transgenic *Prunus* rootstocks have been published (Sabbadini et al., 2015; Song, 2015) and at least a couple of host susceptibility gene classes have been characterized (Decroocq et al., 2006; Huang et al., 2010).

Finally, the last but not least research activity with a great impact for the exploitation of PPV resistance strategies is the development of robust genotype-independent stone fruits transformation-regeneration protocols.

### Conflict of Interest Statement

The authors declare that the research was conducted in the absence of any commercial or financial relationships that could be construed as a potential conflict of interest.

## Footnotes

1 http://faostat3.fao.org

2 http://www.eppo.int/quarantine/quarantine.htm

3 http://www.pestalert.org/opr_search.cfm

4 http://www.foodstandards.gov.au

5 http://www.rosaceae.org/species/prunus_persica/genome_v2.0.a1

## References

[B1] Axtell M. J. (2013). Classification and comparison of small RNAs from plants. Ann. Rev. Plant Biol. 64, 137–159. 10.1146/annurev-arplant-050312-120043 23330790

[B2] Baltes N. J. Gil-Humanes J. Cermak T. Atkins P. A. Voytas D. F. (2014). DNA replicons for plant genome engineering. Plant Cell 26, 151–163. 10.1105/tpc.113.119792 24443519 PMC3963565

[B3] Barajas D. Tenllado F. Gonzales-Jara P. Martinez-Garcia B. Atencio F. A. Diaz-Ruiz J. R. (2004). Resistance to *Plum pox virus* (PPV) in *Nicotiana benthamiana* plants trasformed with the PPV HC-Pro silencing suppressor gene. J. Plant Pathol. 86, 239–248.

[B4] Barba M. Ilardi V. Pasquini G. (2015). Control of pome and stone fruit virus diseases. Adv. Virus Res. 91, 47–83. 10.1016/bs.aivir.2014.11.001 25591877

[B5] Bau H. J. Cheng Y. H. Yu T. A. Yang J. S. Yeh S. D. (2003). Broad-spectrum resistance to different geographic strains of *Papaya ringspot virus* in coat protein gene transgenic papaya. Phytopathologische 93, 112–120. 10.1094/PHYTO.2003.93.1.112 18944164

[B6] Baulcombe D. (2004). RNA silencing in plants. Nature 431, 356–363. 10.1038/nature02874 15372043

[B7] Bendahmane A. Kanyuka K. Baulcombe D. C. (1999). The Rx gene from potato controls separate virus resistance and cell death responses. Plant Cell 11, 781–791. 10330465 10.1105/tpc.11.5.781 PMC144223

[B8] Besford R. T. Hand P. Richardson C. M. Peppitt S. D. (1996). Photoperiod effect on bud burst in *Prunus* is phase dependent: significance for early photosynthetic development. Tree Physiol. 16, 491–496. 14871718 10.1093/treephys/16.5.491

[B9] Bitinaite J. Wah D. A. Aggarwal A. K. Schildkraut I. (1998). FokI dimerization is required for DNA cleavage. Proc. Natl. Acad. Sci. U.S.A. 95, 10570–10575. 9724744 10.1073/pnas.95.18.10570 PMC27935

[B10] Bologna N. G. Voinnet O. (2014). The diversity, biogenesis, and activities of endogenous silencing small RNAs in Arabidopsis. Ann. Rev. Plant Biol. 65, 473–503. 10.1146/annurev-arplant-050213-035728 24579988

[B11] Boonrod K. Galetzka D. Nagy P. D. Conrad U. Krczal G. (2004). Single-chain antibodies against a plant viral RNA-dependent RNA polymerase confer virus resistance. Nat. Biotech. 22, 856–862. 10.1038/nbt983 15195103

[B12] Bosque G. Folch-Fortuny A. Picó J. Ferrer A. Elena S. F. (2014). Topology analysis and visualization of Potyvirus protein-protein interaction network. BMC Syst. Biol. 8:129. 10.1186/s12918-014-0129-8 25409737 PMC4251984

[B13] Brodersen P. Voinnet O. (2006). The diversity of RNA silencing pathways in plants. Trends Genet. 22, 268–280. 10.1016/j.tig.2006.03.003 16567016

[B14] CABI datasheet. (2014). CABI Datasheet on Plum Pox Virus (PPV). Available at: http://www.cabi.org/isc/datasheet/42203

[B15] Cambra M. Capote N. Myrta A. Llácer G. (2006). *Plum pox virus* and the estimated costs associated with sharka disease. EPPO Bull. 36, 202–204. 10.1111/j.1365-2338.2006.01027.x

[B16] Carrasco J. L. Castelló M. J. Naumann K. Lassowskat I. Navarrete-Gómez M. Scheel D. (2014). *Arabidopsis* protein phosphatase DBP1 nucleates a protein network with a role in regulating plant defense. PLoS ONE 9: e0090734. 10.1371/journal.pone.0090734 24595057 PMC3942490

[B17] Castelló M. J. Carrasco J. L. Vera P. (2010). DNA-binding protein phosphatase AtDBP1 mediates susceptibility to two potyviruses in *Arabidopsis*. Plant Physiol. 153, 1521–1525. 10.1104/pp.110.158923 20508138 PMC2923898

[B18] Cavatorta J. Perez K. W. Gray S. M. Van Eck J. Yeam I. Jahn M. (2011). Engineering virus resistance using a modified potato gene. Plant Biotech. J. 9, 1014–1021. 10.1111/j.1467-7652.2011.00622.x 21668622

[B19] Chisholm S. T. Parra M. A. Anderberg R. J. Carrington J. C. (2001). *Arabidopsis* RTM1 and RTM2 genes function in phloem to restrict long-distance movement of Tobacco etch virus. Plant Physiol. 127, 1667–1675. 10.1104/pp.010479 11743111 PMC133571

[B20] Comes S. Fanigliulo A. Piazzolla P. Crescenzi A. (2002). *Nicotiana benthamiana* plants transgenic for PPV-SWC coat protein are resistant to PPV infection. Plant Protec. Sci. 38, 608–611.

[B21] Cornu T. I. Thibodeau-Beganny S. Guhl E. Alwin S. Eichtinger M. Joung J. K. (2008). DNA-binding specificity is a major determinant of the activity and toxicity of zinc-finger nucleases. Mol. Ther. 16, 352–358. 10.1038/sj.mt.6300357 28178540

[B22] Cosson P. Sofer L. Le Q. H. Léger V. Schurdi-Levraud V. Whitham S. A. (2010). RTM3, which controls long-distance movement of potyviruses, is a member of a new plant gene family encoding a meprin and TRAF homology domain-containing protein. Plant Physiol. 154, 222–232. 10.1104/pp.110.155754 20584941 PMC2938151

[B23] Cosson P. Schurdi-Levraud V. Le Q. H. Sicard O. Caballero M. Roux F. (2012). The RTM resistance to potyviruses in *Arabidopsis thaliana*: natural variation of the RTM genes and evidence for the implication of additional genes. PLoS ONE 6:e39169. 10.1371/journal.pone.0039169 22723957 PMC3377653

[B24] Cox D. B. T. Platt R. J. Zhang F. (2015). Therapeutic genome editing: prospects and challenges. Nat. Med. 21, 121–131. 10.1038/nm.3793 25654603 PMC4492683

[B25] Csorba T. Kontra L. Burgyán J. (2015). Viral silencing suppressors: tools forged to fine-tune host-pathogen coexistence. Virology 479–480, 85–103. 10.1016/j.virol.2015.02.028 25766638

[B26] Dangl J. L. Jones J. D. (2001). Plant pathogens and integrated defence responses to infection. Nature 411, 826–833. 10.1038/35081161 11459065

[B27] Decroocq V. Salvador B. Sicard O. Glasa M. Cosson P. Svanella-Dumas L. (2009). The determinant of potyvirus ability to overcome the RTM resistance of *Arabidopsis thaliana* maps to the N-terminal region of the coat protein. Mol. Plant Microbe Interact. 22, 1302–1311. 10.1094/MPMI-22-10-1302 19737103

[B28] Decroocq V. Sicard O. Alamillo J. M. Lansac M. Eyquard J. P. Garcia J. A. (2006). Multiple resistance traits control *Plum pox virus* infection in *Arabidopsis thaliana*. Mol. Plant Microbe Interact. 19, 541–549. 10.1094/MPMI-19-0541 16673941

[B29] Decroocq S. Chague A. Lambert P. Roch G. Audergon J. M. Geuna F. (2014). Selecting with markers linked to the PPVres major QTL is not sufficient to predict resistance to *Plum pox virus* (PPV) in apricot. Tree Genet. Genomes 10, 1161–1170. 10.1007/s11295-014-0750-0

[B30] Di Nicola-Negri E. Brunetti A. Tavazza M. Ilardi V. (2005). Hairpin RNAmediated silencing of *Plum pox virus* P1 and HC-Pro genes for efficient and predictable resistance to the virus. Transgenic Res. 14, 989–994. 10.1007/s11248-005-1773-y 16315100

[B31] Di Nicola-Negri E. Tavazza M. Salandri L. Ilardi V. (2010). Silencing of *Plum pox virus* 5′ UTR/P1 sequence confers resistance to a wide range of PPV strains. Plant Cell Rep. 29, 1435–1444. 10.1007/s00299-010-0933-6 20963442

[B32] Di Nicola E. Tavazza M. Lucioli A. Salandri L. Ilardi V. (2014). Robust RNA silencing-mediated resistance to *Plum pox virus* under variable abiotic and biotic conditions. Mol. Plant Path. 15, 841–847. 10.1111/mpp.12132 25346969 PMC6638643

[B33] Ding S. W. Voinnet O. (2007). Antiviral immunity directed by small RNAs. Cell 130, 413–426. 10.1016/j.cell.2007.07.039 17693253 PMC2703654

[B34] Dolgov S. Mikhaylov R. Serova T. Shulga O. Firsov A. (2010). Pathogen-derived methods for improving resistance of transgenic plums (*Prunus domestica* L.) for *Plum pox virus* infection. Julius Kühn Archiv 427, 133–140.

[B35] Dondini L. Lain O. Vendramin V. Rizzo M. Vivoli D. Adami M. (2011). Identification of QTL for resistance to *Plum pox virus* strains M and D in Lito and Harcot apricot cultivars. Mol. Breed. 27, 289–299. 10.1007/s11032-010-9431-3

[B36] Duprat A. Caranta C. Revers F. Menand B. Browning K. S. Robaglia C. (2002). The *Arabidopsis* eukaryotic initiation factor (iso) 4E is dispensable for plant growth but required for susceptibility to potyviruses. Plant J. 32, 927–934. 10.1046/j.1365-313X.2002.01481.x 12492835

[B37] EFSA panel on Genetically Modified Organisms GMO. (2012). Scientific opinion addressing the safety assessment of plants developed through cisgenesis and intragenesis. EFSA J. 10, 1–33. 10.2903/j.efsa.2012.2561

[B38] Elena S. F. Rodrigo G. (2012). Towards an integrated molecular model of plant–virus interactions. Curr. Opin. Virol. 2, 719–724. 10.1016/j.coviro.2012.09.004 23017245

[B39] Estevan J. Maréna A. Callot C. Lacombe S. Moretti A. Caranta C. (2014). Specific requirement for translation initiation factor 4E or its isoform drives plant host susceptibility to Tobacco etch virus. BMC Plant Biol. 14:67. 10.1186/1471-2229-14-67 24645730 PMC3999954

[B40] Fahim M. Millar A. A. Wood C. C. Larkin P. J. (2012). Resistance to Wheat streak mosaic virus generated by expression of an artificial polycistronic microRNA in wheat. Plant Biotech. J. 10, 150–163. 10.1111/j.1467-7652.2011.00647.x 21895944

[B41] Faino L. Thomma B. P. (2014). Get your high-quality low-cost genome sequence. Trends Plant Sci. 19, 288–291. 10.1016/j.tplants.2014.02.003 24636622

[B42] Farnham G. Baulcombe D. C. (2006). Artificial evolution extends the spectrum of viruses that are targeted by a disease-resistance gene from potato. Proc. Natl. Acad. Sci. U.S.A. 103, 18828–18833. 10.1073/pnas.0605777103 17021014 PMC1693747

[B43] Feng Z. Mao Y. Xu N. Zhang B. Wei P. Yang D. L. (2014). Multigeneration analysis reveals the inheritance, specificity, and patterns of CRISPR/Cas-induced gene modifications in *Arabidopsis*. Proc. Natl. Acad. Sci. U.S.A. 111, 4632–4637. 10.1073/pnas.1400822111 24550464 PMC3970504

[B44] Fichtner F. Castellanos R. U. Ülker B. (2014). Precision genetic modifications: a new era in molecular biology and crop improvement. Planta 239, 921–939. 10.1007/s00425-014-2029-y 24510124

[B45] Gaj T. Gersbach C. A. Barbas C. F. (2013). ZFN, TALEN, and CRISPR/Cas-based methods for genome engineering. Trends Biotechnol. 31, 397–405. 10.1016/j.tibtech.2013.04.004 23664777 PMC3694601

[B46] García J. A. Glasa M. Cambra M. Candresse T. (2014). *Plum pox virus* and sharka: a model potyvirus and a major disease. Mol. Plant Pathol. 15, 226–241. 10.1111/mpp.12083 24102673 PMC6638681

[B47] García-Almodóvar R. C. Clemente-Moreno M. J. Díaz-Vivancos P. Petri C. Rubio M. Padilla I. M. G. (2015). Greenhouse evaluation confirms *in vitro* sharka resistance of genetically engineered h-UTR/P1 plum plants. Plant Cell Tiss. Org. 120, 791–796. 10.1007/s11240-014-0629-7

[B48] García-Gusano M. Martínez-García P. J. Dicenta F. (2010). Seed germination time as a criterion for the early selection of late-flowering almonds. Plant Breed. 129, 578–580. 10.1111/j.1439-0523.2009.01726.x

[B49] Gil M. Esteban O. García J. A. Peña L. Cambra M. (2011). Resistance to *Plum pox virus* in plants expressing cytosolic and nuclear single-chain antibodies against the viral RNA NIb replicase. Plant Pathol. 60, 967–976. 10.1111/j.1365-3059.2011.02448.x

[B50] Guo H. S. Cervera M. T. Garcia J. A. (1998). Plum pox Potyvirus resistance associated to transgene silencing that can be stabilized after different number of plant generations. Gene 206, 263–272. 10.1016/S0378-1119(97)00595-7 9469941

[B51] Guo H. S. Garcia J. A. (1997). Delayed resistance to Plum pox Potyvirus mediated by a mutated RNA replicase gene: involvement of a gene-silencing mechanism. Mol. Plant Micr. Inter. 10, 160–170.

[B52] Händel E. M. Alwin S. Cathomen T. (2009). Expanding or restricting the target site repertoire of zinc-finger nucleases: the inter-domain linker as a major determinant of target site selectivity. Mol. Ther. 17, 104–111. 10.1038/mt.2008.233 19002164 PMC2834978

[B53] Hansche P. E. (1986). Heritability of juvenility in peach. Hort. Sci. 21, 1197–1198.

[B54] Harris C. J. Slootweg E. J. Goverse A. Baulcombe D. C. (2013). Stepwise artificial evolution of a plant disease resistance gene. Proc. Natl. Acad. Sci. U.S.A. 110, 21189–21194. 10.1073/pnas.1311134110 24324167 PMC3876221

[B55] Hartmann W. Neumuller M. (2009). “Plum breeding,” in Breeding Plantation Tree Crops: Temperate Species, eds Mohan J. S. Priyadarshan P. M. (New York: Springer), 161–231. 10.1007/978-0-387-71203-1_6

[B56] Hily J. M. Ravelonandro M. Damsteegt V. Bassett C. Petri C. Liu Z. (2007). *Plum pox virus* coat protein gene intron hairpin RNA construct provides resistance to *Plum pox virus* in *Nicotiana benthamiana* and *Prunus domestica*. J. Am. Soc. Hort. Sci. 132, 850–858.

[B57] Holme I. B. Wendt T. Holm P. B. (2013). Intragenesis and cisgenesis as alternatives to transgenic crop development. Plant Biotech. J. 11, 395–407. 10.1111/pbi.12055 23421562

[B58] Huang T. S. Wei T. Laliberté J. F. Wang A. (2010). A host RNA helicase-like protein, AtRH8, interacts with the potyviral genome-linked protein, VPg, associates with the virus accumulation complex, and is essential for infection. Plant Physiol. 152, 255–266. 10.1104/pp.109.147983 19880609 PMC2799361

[B59] Ilardi V. Di Nicola-Negri E. (2011). Genetically engineered resistance to *Plum pox virus* infection in herbaceous and stone fruit hosts. GM Crops 2, 24–33. 10.4161/gmcr.2.1.15096 21844696

[B60] Jackson A. L. Bartz S. R. Schelter J. Kobayashi S. V. Burchard J. Mao M. (2003). Expression profiling reveals off-target gene regulation by RNAi. Nat. Biotech. 21, 635–637. 10.1038/nbt831 12754523

[B61] Jackson R. J. Hellen C. U. Pestova T. V. (2010). The mechanism of eukaryotic translation initiation and principles of its regulation. Nat. Rev. Mol. Cell Biol. 11, 113–127. 10.1038/nrm2838 20094052 PMC4461372

[B62] Jacquet C. Ravelonandro M. Bachelier J. C. Dunez J. (1998). High resistance to *Plum pox virus* (PPV) in transgenic plants containing modified and truncated form of PPV coat protein gene. Trans. Res. 7, 29–39.

[B63] Jiang F. Song Y. Z. Han Q. J. Zhu C. X. Wen F. J. (2011). The choice of target site is crucial in artificial miRNA-mediated virus resistance in transgenic *Nicotiana tabacum*. Physiol. Mol. Plant Pathol. 76, 2–8. 10.1016/j.pmpp.2011.07.002

[B64] Jiang W. Zhou H. Bi H. Fromm M. Yang B. Weeks D. P. (2013). Demonstration of CRISPR/Cas9/sgRNA-mediated targeted gene modification in *Arabidopsis*, tobacco, sorghum and rice. Nucleic Acids Res. 41:e188. 10.1093/nar/gkt780 23999092 PMC3814374

[B65] Jiménez I. López L. Alamillo J. M. Valli A. García J. A. (2006). Identification of a *Plum pox virus* CI-interacting protein from chloroplast that has a negative effect in virus infection. Mol. Plant Microbe Interact. 19, 350–358. 10.1094/MPMI-19-0350 16570664

[B66] Kasschau K. D. Xie Z. X. Allen E. Llave C. Chapman E. J. Krizan K. A. (2003). P1/HC-Pro, a viral suppressor of RNA silencing, interferes with *Arabidopsis* development and miRNA function. Dev. Cell 4, 205–217. 10.1016/S1534-5807(03)00025-X 12586064

[B67] Knoll A. Fauser F. Puchta H. (2014). DNA recombination in somatic plant cells: mechanisms and evolutionary consequences. Chrom. Res. 22, 191–201. 10.1007/s10577-014-9415-y 24788060

[B68] Kung Y. J. Lin S. S. Huang Y. L. Chen T. C. Harish S. S. Chua N. H. (2012). Multiple artificial microRNAs targeting conserved motifs of the replicase gene confer robust transgenic resistance to negative-sense single-stranded RNA plant virus. Mol. Plant Pathol. 13, 303–317. 10.1111/j.1364-3703.2011.00747.x 21929564 PMC6638711

[B69] Lafforgue G. Martínez F. Niu Q. W. Chua N. H. Daròs J. A. Elena S. F. (2013). Improving the effectiveness of artificial microRNA (amiR)-mediated resistance against Turnip mosaic virus by combining two amiRs or by targeting highly conserved viral genomic regions. J. Virol. 87, 8254–8256. 10.1128/JVI.00914-13 23698292 PMC3700214

[B70] Lafforgue G. Martínez F. Sardanyés J. de la Iglesia F. Niu Q. W. Lin S. S. (2011). Tempo and mode of plant RNA virus escape from RNA interference mediated resistance. J. Virol. 85, 9686–9695. 10.1128/JVI.05326-11 21775453 PMC3196453

[B71] Laimer da Camara Machado M. da Camara A. Hanzer V. Weiss H. Regner F. Steinkellner H. (1992). Regeneration of transgenic plants of *Prunus armeniaca* containing the coat protein gene of *Plum pox virus*. Plant Cell Rep. 11, 25–29. 24213032 10.1007/BF00231834

[B72] Lellis A. D. Kasschau K. D. Whitham S. A. Carrington J. C. (2002). Loss-of-susceptibility mutants of *Arabidopsis thaliana* reveal an essential role for eIF (iso) 4E during potyvirus infection. Curr. Biol. 12, 1046–1051. 10.1016/S0960-9822(02)00898-9 12123581

[B73] Léonard S. Plante D. Wittmann S. Daigneault N. Fortin M. G. Laliberté J. F. (2000). Complex formation between potyvirus VPg and translation eukaryotic initiation factor 4E correlates with virus infectivity. J. Virol. 74, 7730–7737. 10.1128/JVI.74.17.7730-7737.2000 10933678 PMC112301

[B74] Li J. F. Norville J. E. Aach J. McCormack M. Zhang D. Bush J. (2013). Multiplex and homologous recombination-mediated genome editing in *Arabidopsis* and *Nicotiana benthamiana* using guide RNA and Cas9. Nat. Biotechnol. 31, 688–691. 10.1038/nbt.2654 23929339 PMC4078740

[B75] Li T. Liu B. Spalding M. H. Weeks D. P. Yang B. (2012). High-efficiency TALEN-based gene editing produces disease-resistant rice. Nat. Biotechnol. 30, 390–392. 10.1038/nbt.2199 22565958

[B76] Lin S. Wu H. Elena S. Chen K. Niu Q. Ye S. (2009). Molecular evolution of a viral non-coding sequence under the selective pressure of amiRNA-mediated silencing. PLoS Pathog. 5:e1000312. 10.1371/journal.ppat.1000312 19247440 PMC2642722

[B77] Liu Z. Scorza R. Hily J. M. Scott S. W. James D. (2007). Engineering resistance to multiple *Prunus* fruit viruses through expression of chimeric hairpins. J. Am. Soc. Hort. Sci. 132, 407–414.

[B78] López-Noguera S. Petri C. Burgos L. (2009). Combining a regeneration-promoting ipt gene and site-specific recombination allows a more efficient apricot transformation and the elimination of marker genes. Plant Cell Rep. 28, 1781–1790. 10.1007/s00299-009-0778-z 19820947

[B79] Lu S. Shi R. Tsao C. Yi X. Li L. Chiang V. L. (2004). RNA silencing in plants by the expression of siRNA duplexes. Nucleic Acids Res. 32, e171. 10.1093/nar/gnh170 15576678 PMC535699

[B80] Maki-Valkama T. Valkonen J. P. T. Kreuze J. F. Pehu E. (2000). Transgenic resistance to PVYO associated with post-transcriptional silencing of P1 transgene is overcome by PVYN strains that carry highly homologous P1 sequences and recover transgene expression at infection. Mol. Plant Microbe Interact. 13, 66–373. 10.1094/MPMI.2000.13.4.366 10755299

[B81] Malinowski T. Cambra M. Capote N. Zawadzka B. Gorris M. T. Scorza R. (2006). Field trials of plum clones transformed with the *Plum pox virus* coat protein (PPVCP) gene. Plant Disease 90, 1012–1018. 10.1094/PD-90-1012 30781292

[B82] Mallory A. C. Vaucheret H. (2006). Functions of microRNAs and related small RNAs in plants. Nature Genet. 38, S31–S36. 10.1038/ng1791 16736022

[B83] Marandel G. Salava J. Abbott A. Candresse T. Decroocq V. (2009). Quantitative trait loci meta-analysis of *Plum pox virus* resistance in apricot (*Prunus armeniaca* L.)*:* new insights on the organization and the identification of genomic resistance factors. Mol. Plant Pathol. 10, 347–360. 10.1111/J.1364-3703.2009.00535.X 19400838 PMC6640416

[B84] Martínez F. Elena S. F. Daròs J. A. (2013). Fate of artificial microRNA-mediated resistance to plant viruses in mixed infections. Phytopathologische 103, 870–876. 10.1094/PHYTO-09-12-0233-R 23617337

[B85] Martínez F. Lafforgue G. Morelli M. J. González-Candelas F. Chua N. H. Daròs J. A. (2012). Ultradeep sequencing analysis of population dynamics of virus escape mutants in RNAi-mediated resistant plants. Mol. Biol. Evol. 29, 3297–3307. 10.1093/molbev/mss135 22593223 PMC7187544

[B86] Marton I. Zuker A. Shklarman E. Zeevi V. Tovkach A. Roffe S. (2010). Nontransgenic genome modification in plant cells. Plant Physiol. 154, 1079–1087. 10.1104/pp.110.164806 20876340 PMC2971589

[B87] Matzke M. A. Mosher R. A. (2014). RNA-directed DNA methylation: an epigenetic pathway of increasing complexity. Nature Rev. Genet. 15, 394–408. 10.1038/nrg3683 24805120

[B88] Meckler J. F. Bhakta M. S. Kim M. S. Ovadia R. Habrian C. H. Zykovich A. (2013). Quantitative analysis of TALE–DNA interactions suggests polarity effects. Nucleic Acids Res. 41, 4118–4128. 10.1093/nar/gkt085 23408851 PMC3627578

[B89] Miller J. C. Tan S. Qiao G. Barlow K. A. Wang J. Xia D. F. (2011). A TALE nuclease architecture for efficient genome editing. Nat. Biotechnol. 29, 143–148. 10.1038/nbt.1755 21179091

[B90] Monticelli S. Di Nicola-Negri E. Gentile A. Damiano C. Ilardi V. (2012). Production and *in vitro* assessment of transgenic plums for resistance to *Plum pox virus*: a feasible, environmental risk-free, cost-effective approach. Ann. Appl. Biol. 161, 293–301. 10.1111/j.1744-7348.2012.00573.x

[B91] Moreno A. Fereres A. Cambra M. (2009). Quantitative estimation of *Plum pox virus* targets acquired and transmitted by a single *Myzus persicae*. Arch. Virol. 154, 1391–1399. 10.1007/s00705-009-0450-5 19597934

[B92] Mourrain P. Béclin C. Elmayan T. Feuerbach F. Godon C. Morel J. B. (2000). *Arabidopsis* SGS2 and SGS3 genes are required for posttranscriptional gene silencing and natural virus resistance. Cell 101, 533–542. 10.1016/S0092-8674(00)80863-6 10850495

[B93] Mussolino C. Morbitzer R. Lütge F. Dannemann N. Lahaye T. Cathomen T. (2011). A novel TALE nuclease scaffold enables high genome editing activity in combination with low toxicity. Nucleic Acids Res. 39, 9283–9293. 10.1093/nar/gkr597 21813459 PMC3241638

[B94] Nekrasov V. Staskawicz B. Weigel D. Jones J. D. Kamoun S. (2013). Targeted mutagenesis in the model plant *Nicotiana benthamiana* using Cas9 RNA-guided endonuclease. Nat. Biotechnol. 31, 691–693. 10.1038/nbt.2655 23929340

[B95] Niu Q. W. Lin S. S. Reyes J. L. Chen K. C. Wu H. W. Yeh S. D. (2006). Expression of artificial microRNAs in transgenic *Arabidopsis thaliana* confers virus resistance. Nat. Biotechnol. 24, 1420–1428. 10.1038/nbt1255 17057702

[B96] Osakabe K. Osakabe Y. Toki S. (2010). Site-directed mutagenesis in *Arabidopsis* using custom-designed zinc finger nucleases. Proc. Natl. Acad. Sci. U.S.A. 107, 12034–12039. 10.1073/pnas.1000234107 20508151 PMC2900650

[B97] Palkovics L. Wittner A. Balazs E. (1995). Pathogen-derived resistance induced by integrating the *Plum pox virus* coat protein gene into plants of *Nicotiana benthamiana*. Acta Hort. 386, 311–317.

[B98] Pandolfini T. Molesini B. Avesani L. Spena A. Polverari A. (2003). Expression of self-complementary hairpin RNA under the control of the rolC promoter confers systemic disease resistance to *Plum pox virus* without preventing local infection. BMC Biotech. 3:7. 10.1186/1472-6750-3-7 12823862 PMC194883

[B99] Pasquini G. Barba M. (2006). The question of seed transmissibility of *Plum pox virus*. EPPO Bull. 36, 287–292. 10.1111/j.1365-2338.2006.00989.x

[B100] Perring T. M. Gruenhagen T. M. Farrar C. A. (1999). Management of plant viral diseases through chemical control of insect. Ann. Rev. Entomol. 44, 457–481. 10.1146/annurev.ento.44.1.457 15012379

[B101] Petri C. Alburquerque N. Burgos L. (2015). “Apricot (*Prunus armeniaca* L.)” in Agrobacterium Protocols, ed. Wang K. (New York: Springer), 111–119. 10.1007/978-1-4939-1658-0_10 25416253

[B102] Petri C. Burgos L. (2005). Transformation of fruit trees. Useful breeding tool or continued future prospect? Trans. Res. 14, 15–26. 10.1007/s11248-004-2770-2 15865045

[B103] Petri C. Hily J. M. Vann C. Dardick C. Scorza R. (2011). A high-throughput transformation system allows the regeneration of marker-free plum plants (*Prunus domestica*). Ann. Appl. Biol. 159, 302–315. 10.1111/j.1744-7348.2011.00499.x

[B104] Petri C. López-Noguera S. Wang H. García-Almodóvar C. Alburquerque N. Burgos L. (2012). A chemical-inducible Cre-LoxP system allows for elimination of selection marker genes in transgenic apricot. Plant Cell Tiss. Org. 110, 337–346. 10.1007/s11240-012-0155-4

[B105] Petri C. Webb K. Hily J. M. Dardick C. Scorza R. (2008). High transformation efficiency in plum (*Prunus domestica* L.): a new tool for functional genomics studies in *Prunus* spp. Mol. Breed. 22, 581–591. 10.1007/s11032-008-9200-8

[B106] Polak J. Pivalova J. Kundu J. K. Jokes M. Scorza R. Ravelonandro M. (2008). Behaviour of transgenic *Plum pox virus* resistant *Prunus domestica* L. *clone C*5 grown in the open field under a high and permanent infection pressure of the PPV-Rec strain. J. Plant Pathol. 90, 33–36.

[B107] Potter D. (2012). “Basic information on the stone fruit crops,” in Genetics, Genomics and Breeding of Stone Fruits, eds. Kole C. Abbott A.G. (New York: CRC), 1–21.

[B108] Pruett-Miller S. M. Reading D. W. Porter S. N. Porteus M. H. (2009). Attenuation of zinc finger nuclease toxicity by small-molecule regulation of protein levels. PLoS Genet. 5:e1000376. 10.1371/journal.pgen.1000376 19214211 PMC2633050

[B109] Qu F. Ye X. Morris T. J. (2008). *Arabidopsis* DRB4, AGO1, AGO7, and RDR6 participate in a DCL4-initiated antiviral RNA silencing pathway negatively regulated by DCL1. Proc. Natl. Acad. Sci. U.S.A. 105, 732–737. 10.1073/pnas.0805760105 18799732 PMC2567185

[B110] Qu J. Ye J. Fang R. (2007). Artificial microRNA-mediated virus resistance in plants. J. Virol. 81, 6690–6699. 10.1128/JVI.02457-06 17344304 PMC1900123

[B111] Ramesh S. A. Kaiser B. N. Franks T. Collins G. Sedgley M. (2006). Improved methods in Agrobacterium–mediated transformation of almond using positive (mannose/pmi) or negative (kanamycin resistance) selection-based protocols. Plant Cell Rep. 25, 821–828. 10.1007/s00299-006-0139-0 16534597

[B112] Ramirez C. L. Foley J. E. Wright D. A. Müller-Lerch F. Rahman S. H. Cornu T. I. (2008). Unexpected failure rates for modular assembly of engineered zinc fingers. Nat. Meth. 5, 374–375. 10.1038/nmeth0508-374 18446154 PMC7880305

[B113] Ravelonandro M. Briard P. Hily J. M. Scorza R. Lomberk D. (2015). Evaluation of *Plum pox virus* (PPV) CP and P1 constructs on sharka resistance in plum (*Prunus domestica*). Acta Hort. 1063, 63–70.

[B114] Ravelonandro M. Monsion M. Teycheney P. Y. Delbos R. Dunez J. (1992). Construction of a chimeric viral gene expressing *Plum pox virus* coat protein. Gene 120, 167–173. 1398133 10.1016/0378-1119(92)90090-c

[B115] Ravelonandro M. Scorza R. Hily J. M. Briard P. (2014). The efficiency of RNA interference for conferring stable resistance to *Plum pox virus*. Plant Cell Tiss. Organ Cult. 118, 347–356. 10.1007/s11240-014-0487-3

[B116] Regner F. da Camera Machado A. Laimer da Camera Machado M. Steinkellner H. Mattanovich D. Hanzer V. (1992). Coat protein mediated resistance to *Plum pox virus* in *Nicotiana clevelandii* and *N. benthamiana*. Plant Cell Rep. 11, 30–33. 24213033 10.1007/BF00231835

[B117] Revers F. García J. A. (2015). Molecular biology of Potyviruses. Adv. Virus Res. 92, 101–199. 10.1016/bs.aivir.2014.11.006 25701887

[B118] Rodamilans B. San León D. Mühlberger L. Candresse T. Neumüller M. Oliveros J. C. (2014). Transcriptomic analysis of *Prunus domestica* undergoing hypersensitive response to *Plum pox virus* infection. PLoS ONE 9:e100477. 10.1371/journal.pone.0100477 24959894 PMC4069073

[B119] Rommens C. M. (2004). All-native DNA transformation: a new approach to plant genetic engineering. Trends Plant Sci. 9, 457–464. 10.1016/j.tplants.2004.07.001 15337496

[B120] Rubio M. Martínez-Gómez P. Dicenta F. Weber W. E. (2003). Resistance of almond cultivars to *Plum pox virus* (sharka). Plant Breed. 122, 462–464. 10.1046/j.1439-0523.2003.00872.x

[B121] Rubio M. Martínez-Gómez P. García J. A. Dicenta F. (2013). Interspecific transfer of resistance to *Plum pox virus* from almond to peach by grafting. Ann. Appl. Biol. 163, 466–474. 10.1111/aab.12069

[B122] Rubio M. Pascal T. Bachellez A. Lambert P. (2010). Quantitative trait loci analysis of *Plum pox virus* resistance in Prunus davidiana P1908: new insights on the organization of genomic resistance regions. Tree Genet. Genomes 6, 291–304. 10.1007/s11295-009-0249-2

[B123] Rubio M. Ruiz D. Egea J. Martínez-Gómez P. Dicenta F. (2014). Opportunities of marker-assisted selection for *Plum pox virus* resistance in apricot breeding programs. Tree Genet. Genom. 10, 513–525. 10.1007/s11295-014-0700-x

[B124] Ruffel S. Dussault M. H. Palloix A. Moury B. Bendahmane A. Robaglia C. (2002). A natural recessive resistance gene against Potato virus Y in pepper corresponds to the eukaryotic initiation factor 4E (eIF4E). Plant J. 32, 1067–1075. 10.1038/sj.embor.7400769 12492847

[B125] Sabbadini S. Pandolfini T. Girolomini L. Molesini B. Navacchi O. (2015). “Peach (*Prunus persica* L.)” in Agrobacterium Protocols, ed. Wang K. (New York: Springer), 205–215. 10.1007/978-1-4939-1658-0_17 25416260

[B126] Sanford J. C. Johnston S. A. (1985). The concept of parasite-derived resistance. Deriving resistance genes from the parasite’s own genome. J. Theoret. Biol. 113, 395–405. 10.1016/S0022-5193(85)80234-4

[B127] Schouten H. J. Krens F. A. Jacobsen E. (2006). Cisgenic plants are similar to traditionally bred plants. EMBO Rep 7, 750–753. 10.1038/sj.embor.7400769 16880817 PMC1525145

[B128] Scorza R. Callahan A. Dardick C. Ravelonandro M. Polak J. Malinowski T. (2013a). Genetic engineering of *Plum pox virus* resistance: ‘HoneySweet’ plum—from concept to product. Plant Cell Tiss. Organ. Cult. 115, 1–12. 10.1007/s11240-013-0339-6

[B129] Scorza R. Kriss A. B. Callahan A. M. Webb K. Demuth M. Gottwald T. (2013b). Spatial and temporal assessment of pollen-and seed-mediated gene flow from genetically engineered plum *Prunus domestica*. PLoS ONE 8:e75291. 10.1371/journal.pone.0075291 24098374 PMC3788040

[B130] Scorza R. Callahan A. Levy L. Damsteegt V. Webb K. Ravelonandro M. (2001). Post-transcriptional gene silencing in *Plum pox virus* resistant transgenic European plum containing the Plum pox potyvirus coat protein gene. Trans. Res. 1054, 1–9. 11437277 10.1023/a:1016644823203

[B131] Scorza R. Georgi L. Callahan A. Petri C. Hily J. M. Dardick C. (2010). Hairpin *Plum pox virus* coat protein (hpPPV-CP) structure in ‘HoneySweet’ C5 plum provides PPV resistance when genetically engineered into plum (*Prunus domestica*) seedlings. Julius Kühn Archiv 427, 141–146.

[B132] Scorza R. Ravelonandro M. Callahan A. M. Cordts J. M. Fuchs M. Dunez J. (1994). Transgenic plum (*Prunus domestica* L.) express the *Plum pox virus* coat protein gene. Plant Cell Rep. 14, 18–22. 24194220 10.1007/BF00233291

[B133] Senthil-Kumar M. Mysore K. S. (2011). “Caveat of RNAi in plants: the off-target effect,” in RNAi and Plant Gene Function Analysis, eds. Kodama H. Komamine A. (Chiba: Humana Press), 13–25. 10.1007/978-1-61779-123-9_2 21533683

[B134] Shan Q. Wang Y. Chen K. Liang Z. Li J. Zhang Y. (2013a). Rapid and efficient gene modification in rice and brachypodium using TALENs. Mol. Plant 6, 1365–1368. 10.1093/mp/sss162 23288864 PMC3968307

[B135] Shan Q. Wang Y. Li J. Zhang Y. Chen K. Liang Z. (2013b). Targeted genome modification of crop plants using a CRISPR-Cas system. Nat. Biotechnol. 31, 686–688. 10.1038/nbt.2650 23929338

[B136] Shukla V. K. Doyon Y. Miller J. C. DeKelver R. C. Moehle E. A. Worden S. E. (2009). Precise genome modification in the crop species *Zea mays* using zinc-finger nucleases. Nature 459, 437–441. 10.1038/nature07992 19404259

[B137] Simón-Mateo C. García J. A. (2006). MicroRNA-guided processing impairs plum px virus replication, but the virus readily evolves to escape this silencing mechanism. J. Virol. 80, 2429–2436. 10.1128/JVI.80.5.2429-2436.2006 16474149 PMC1395392

[B138] Smith N. A. Singh S. P. Wang M. B. Stoutjesdijk P. A. Green A. G. Waterhouse P. M. (2000). Gene expression: total silencing by intron-spliced hairpin RNAs. Nature 407, 319–320. 10.1038/35030305 11014180

[B139] Song G. Q. (2015). “Cherry,” in Agrobacterium Protocols, ed. Wang K. (New York: Springer), 133–142. 10.1007/978-1-4939-1658-0_12

[B140] Song G. Q. Sink K. C. Walworth A. E. Cook M. A. Allison R. F. Lang G. A. (2013). Engineering cherry rootstocks with resistance to *Prunus necrotic* ring spot virus through RNAi-mediated silencing. Plant Biotech. J. 11, 702–708. 10.1111/pbi.12060 23521804

[B141] Soriano J. M. Domingo M. L. Zuriaga E. Romero C. Zhebentyayeva T. Abbott A. G. Badenes M. L. (2012). Identification of simple sequence repeat markers tightly linked to *Plum pox virus* resistance in apricot. Mol. Breed. 30, 1017–1026. 10.1007/s11032-011-9685-4

[B142] Srinivasan C. Dardick C. Callahan A. Scorza R. (2012). Plum (*Prunus domestica*) trees transformed with poplar FT1 result in altered architecture, dormancy requirement, and continuous flowering. PLoS ONE 7:e40715. 10.1371/journal.pone.0040715 22859952 PMC3408467

[B143] Szittya G. Silhavy D. Molnar A. Havelda Z. Lovas A. Lakatos L. (2003). Low temperature inhibits RNA silencing-mediated defence by the control of siRNA generation. EMBO J. 22, 633–640. 10.1093/emboj/cdg74 12554663 PMC140757

[B144] Tavladoraki P. Benvenuto E. Trinca S. De Martinis D. Cattaneo A. Galeffi P. (1993). Transgenic plants expressing a functional single-chain Fv antibody are specifically protected from virus attack. Nature 366, 469–472. 10.1038/366469a0 8247156

[B145] Townsend J. A. Wright D. A. Winfrey R. J. Fu F. Maeder M. L. Joung J. K. (2009). High-frequency modification of plant genes using engineered zinc-finger nucleases. Nature 459, 442–445. 10.1038/nature07845 19404258 PMC2743854

[B146] Tavert-Roudet G. Ravelonandro M. Bachelier J. C. Dunez J. (1998). Transgenic *Nicotiana benthamiana* plant containing the P1 gene of *Plum pox virus* are resistant to virus challenge. Eur. J. Plant Pathol. 104, 103–107.

[B147] Truniger V. Aranda M. A. (2009). Recessive resistance to plant viruses. Adv. Virus Res. 75, 119–159. 10.1016/S0065-3527(09)07504-6 20109665

[B148] Usenik V. Kastelec D. Stampar F. Virscek Marn M. (2015). The effect of *Plum pox virus* on chemical composition and fruit quality of plum. J. Agric. Food Chem. 63, 51–60. 10.1021/jf505330t 25495040

[B149] Varrelmann M. Maiss E. (2000). Mutations in the coat protein gene of *Plum pox virus* suppress particle assembly, heterologous encapsidation and complementation in transgenic plants of *Nicotiana benthamiana*. J. Gen. Virol. 81, 567–576. 10675394 10.1099/0022-1317-81-3-567

[B150] Vaucheret H. (2008). Plant argonautes. Trends Plant Sci. 13, 350–358. 10.1016/j.tplants.2008.04.007 18508405

[B151] Verde I. Abbott A. G. Scalabrin S. Jung S. Shu S. Marroni F. (2013). The high-quality draft genome of peach (*Prunus persica*) identifies unique patterns of genetic diversity, domestication and genome evolution. Nature Genet. 45, 487–494. 10.1038/ng.2586 23525075

[B152] Voinnet O. (2009). Origin, biogenesis, and activity of plant microRNAs. Cell 136, 669–687. 10.1016/j.cell.2009.01.046 19239888

[B153] Wah D. A. Bitinaite J. Schildkraut I. Aggarwal A. K. (1998). Structure of FokI has implications for DNA cleavage. Proc. Natl. Acad. Sci. U.S.A. 95, 10564–10569. 9724743 10.1073/pnas.95.18.10564 PMC27934

[B154] Wang X. Kohalmi S. E. Svircev A. Wang A. Sanfaçon H. Tian L. (2013a). Silencing of the host factor eIF(iso)4E gene confers *Plum pox virus* resistance in plum. PLoS ONE 8:e50627. 10.1371/journal.pone.0050627 23382802 PMC3557289

[B155] Wang A. Tian L. Brown D. C. W. Svircev A. M. Stobbs L. W. Sanfaçon H. (2013b). Generation of efficient resistance to *Plum pox virus* (PPV) in *Nicotiana benthamiana* and *Prunus domestica* expressing triple-intron-spanned double-hairpin RNAs simultaneously targeting 5′ and 3′ conserved genomic regions of PPV. Acta Hort. 1063, 77–84.

[B156] Wang A. Krishnaswamy S. (2012). Eukaryotic translation initiation factor 4E-mediated recessive resistance to plant viruses and its utility in crop improvement. Mol. Plant Pathol. 13, 795–803. 10.1111/J.1364-3703.2012.00791.X 22379950 PMC6638641

[B157] Wang A. Tian L. Huang T. S. Brown D. C. W. Svircev A. M. Stobbs L. W. (2009). The development of genetic resistance to *Plum pox virus* in transgenic *Nicotiana benthamiana* and *Prunus domestica*. Acta Hort. 839, 665–672.

[B158] Wittmann S. Chatel H. Fortin M. G. Laliberté J. F. (1997). Interaction of the viral protein genome linked of Turnip mosaic potyvirus with the translational eukaryotic initiation factor (iso) 4E of *Arabidopsis thaliana* using the yeast two-hybrid system. Virology 234, 84–92. 10.1006/viro.1997.8634 9234949

[B159] Wittner A. Palkovics L. Balazs E. (1998). *Nicotiana benthamiana* plants transformed with the *Plum pox virus* helicase gene are resistant to virus infection. Virus Res. 53, 97–103. 10.1016/S0168-1702(97)00133-0 9617773

[B160] Wu X. L. Hou W. C. Wang M. M. Zhu X. P. Li F. Zhang J. D. (2008). RNA silencing-mediated resistance is related to biotic/abiotic stresses and cellular RdRp expression in transgenic tobacco plants. BMB Rep. 41, 376–381. 10.5483/BMBRep.2008.41.5.376 18510868

[B161] Xu L. Song Y. Z. Zhu J. H Guo X. Q. Zhu C. X. Wen F. J. (2009). Conserved sequences of replicase gene-mediated resistance to Potyvirus through RNA silencing. J. Plant Biol. 52, 550–559. 10.1007/s12374-009-9071-5

[B162] Yau Y. Y. Stewart C. N. (2013). Less is more: strategies to remove marker genes from transgenic plants. BMC Biotech. 13:36. 10.1186/1472-6750-13-36 23617583 PMC3689633

[B163] Zagrai I. Capote N. Ravelonandro M. Cambra M. Zagrai L. Scorza R. (2008). *Plum pox virus* silencing of C5 transgenic plums is stable under challenge inoculation with heterologous viruses. J. Plant Pathol. 90, 63–71.

[B164] Zhang X. Li H. Zhang J. Zhang C. Gong P. Ziaf (2011). Expression of artificial microRNAs in tomato confers efficient and stable virus resistance in a cell-autonomous manner. Transgenic Res. 20, 569–581. 10.1007/s11248-010-9440-3 20835923

[B165] Zhang S. C. Tian L. M. Svircev A. Brown D. C. W. Sibbald S. Schneider K. E. (2006). Engineering resistance to *Plum pox virus* (PPV) through the expression of PPV specific hairpin RNAs in transgenic plants. Can. J. Plant Pathol. 28, 263–270. 10.1080/07060660609507295

[B166] Zhang Y. Zhang F. Li X. Baller J. A. Qi Y. Starker C. G. (2013). Transcription activator-like effector nucleases enable efficient plant genome engineering. Plant Physiol. 161, 20–27. 10.1104/pp.112.205179 23124327 PMC3532252

[B167] Zhong S. H. Liu J. Z. Jin H. Lin L. Li Q. Chen Y. (2013). Warm temperatures induce transgenerational epigenetic release of RNA silencing by inhibiting siRNA biogenesis in *Arabidopsis*. Proc. Natl. Acad. Sci. U.S.A. 110, 9171–9176. 10.1073/pnas.1219655110 23686579 PMC3670308

[B168] Zuriaga E. Soriano J. M. Zhebentyayeva T. Romero C. Dardick C. Cañizares J. (2013). Genomic analysis reveals MATH gene (s) as candidate (s) for *Plum pox virus* (PPV) resistance in apricot (*Prunus armeniaca* L.). Mol. Plant Pathol. 14, 663–677. 10.1111/mpp.12037 23672686 PMC6638718

